# A Multisource Hardware Sensing Signal Fusion Network for Robust State Prediction and Anomaly Perception

**DOI:** 10.3390/s26134234

**Published:** 2026-07-03

**Authors:** Yufei Li, Junxian Zhao, Yi Wei, Xichen Wang, Yaqing Yang, Yang Yang, Yan Zhan

**Affiliations:** 1 National School of Development, Peking University, Beijing 100871, China; 2 Artificial Intelligence Research Institute, Tsinghua University, Beijing 100084, China

**Keywords:** multisource hardware sensing, sensor fusion, industrial digital systems, edge computing, cross-source signal alignment

## Abstract

With the rapid development of intelligent manufacturing, edge computing, and industrial and financial–industrial digital systems, large volumes of multisource hardware sensing signals are continuously generated in complex production environments, including environmental, electrical, vibration, network communication, and device operational signals. Owing to the heterogeneity, asynchrony, noise interference, and disturbance sensitivity of these signals, conventional state prediction methods often fail to sufficiently characterize the dynamic response relationships among different sensing sources and cannot maintain stable prediction performance under non-stationary scenarios such as load surges, network congestion, and device anomalies. To address these challenges, a multisource hardware sensing signal fusion network is proposed for the edge-computing and digital production test scenario of an intelligent equipment manufacturing enterprise in Hebei Province, China, with the aim of achieving robust state prediction and anomaly perception in complex digital systems. In the proposed method, environmental sensing, device power, edge-node operation, vibration monitoring, network communication, and system output states are uniformly modeled as multisource engineering sensing signals, and an end-to-end prediction framework is constructed with cross-source sensing signal alignment to facilitate temporal coherence, disturbance-aware residual correction to substantially mitigate disturbance contamination, and context-adaptive fusion. Experimental results show that the proposed method achieves the best performance in the overall state prediction task, with MAE, RMSE, MAPE, and $R^{2}$ reaching 0.0968, 0.1457, 8.12%, and 0.9416, respectively, outperforming baseline methods including ARIMA, XGBoost, LightGBM, LSTM, TCN, Transformer, Attention Fusion, and Multimodal Transformer. In the disturbance robustness experiment, the Event-MAE and Event-RMSE of the proposed method are reduced to 0.1126 and 0.1694, respectively, with an Avg. Drop of only 28.98%, indicating that more stable responses can be achieved under non-stationary disturbance scenarios. In the abnormal-state recognition task, Accuracy, Precision, Recall, and F1-score values of 94.32%, 93.76%, 92.85%, and 93.30% are achieved, respectively. The results demonstrate that the proposed method can effectively improve the state prediction accuracy, disturbance robustness, and anomaly warning capability of multisource hardware sensing data in complex industrial and financial–industrial digital systems, thereby providing an effective modeling scheme for intelligent monitoring and engineering decision-making in AI-driven industrial and financial sensing scenarios.

## 1. Introduction

With the rapid development of intelligent manufacturing, the Industrial Internet of Things, edge computing, and industrial and financial–industrial digital systems, large-scale dynamic data streams are continuously generated in complex engineering sites from various hardware sensing devices and system monitoring modules. Unlike traditional single-device monitoring scenarios, modern industrial and financial–industrial digital systems usually involve multiple observation sources, including environmental sensing, equipment electrical states, edge-node operational states, mechanical vibration, network communication states, and system output states. These signals characterize system states from different perspectives, including the physical environment, hardware load, computing resources, mechanical disturbances, communication links, service operations, and transaction-processing infrastructure, thereby forming the multisource hardware sensing data foundation for industrial edge-computing systems [1,2]. For example, environmental signals such as temperature, humidity, illumination, and barometric pressure reflect external operating conditions; voltage, current, and power consumption describe hardware load and energy variation; CPU load, GPU load, memory usage, and device temperature reflect edge-node computing status; vibration signals capture mechanical disturbances and local impacts; and network traffic, response latency, and packet loss rate reflect communication stability and service quality. Although these signals differ in sampling frequency, physical meaning, noise distribution, and response speed, they jointly reflect the dynamic evolution and potential abnormal states of industrial and financial–industrial digital systems. Therefore, unified modeling of multisource hardware sensing signals for robust state prediction and anomaly perception has become an important issue in intelligent sensing, industrial monitoring, financial infrastructure monitoring, and edge-intelligence-based operation and maintenance.

Traditional methods for system-state prediction and anomaly detection mainly rely on a single time series or a small number of structured operational indicators. Early studies adopted statistical models such as ARIMA and GARCH to model equipment states, load variations, or system performance trends [3]. Machine learning methods such as Isolation Forest, support vector machines, and random forests were subsequently applied to anomaly detection and operational-state recognition tasks [4]. In recent years, LSTM, GRU, TCN, and Transformer have demonstrated strong dynamic modeling capabilities in industrial time-series prediction, equipment health monitoring, financial time-series risk perception, and anomaly recognition [5,6]. However, these methods often treat system observations as a single sequence or simply concatenated feature vectors, making it difficult to characterize asynchronous response relationships and dynamic coupling mechanisms among hardware sensing sources. In practical industrial edge-computing systems, environmental changes may first affect device temperature and heat dissipation, then trigger computing-load fluctuations and service latency; network congestion may first appear as traffic peaks and increased packet loss before affecting task completion and service availability; and mechanical vibration may cause hardware-state fluctuations and abnormal alarms within a short period. Therefore, single-source modeling or static feature fusion is insufficient for stable prediction and timely anomaly perception under complex disturbances.

To address these limitations, multisource sensor fusion and multimodal industrial state modeling methods have been increasingly explored. Early studies mainly adopted feature-level fusion, where statistical features from different sensors were concatenated within a unified time window and then fed into machine learning or deep learning models [7,8]. With the development of attention mechanisms and representation learning, attention-based fusion models, graph neural networks, and multimodal Transformers have been used to learn interactions among different sensing sources [9]. However, several challenges remain. First, multisource hardware sensing signals show significant temporal-scale inconsistency. Environmental sensors may sample at minute-level frequencies, and electrical and operational data may be updated at second-level frequencies, whereas vibration signals may be generated at much higher frequencies. Simple time-window aggregation may lose high-frequency disturbance details, while strict timestamp alignment may introduce sparsity, noise, and spurious correlations. Second, leading–lagging response relationships exist among sensing sources. Abnormal events usually do not appear synchronously in all channels, but they gradually propagate along chains such as “environment–device–network–system output”. Similar propagation patterns may also appear in financial–industrial infrastructures, where transaction-processing load, network latency, abnormal access frequency, and service availability jointly reflect infrastructure risk. Third, industrial edge systems are affected by non-stationary disturbances, including load surges, network congestion, restricted heat dissipation, short-term sensor failures, and environmental fluctuations. If long-term stable trends and short-term disturbance responses cannot be distinguished, local anomalies may be incorrectly learned as regular patterns, leading to prediction bias and missed anomaly detection.

Deep learning methods provide new technical pathways for multisource hardware sensing modeling in complex industrial systems. Through temporal convolution, recurrent networks, self-attention mechanisms, and contextual gating structures, nonlinear dynamic features can be extracted from multidimensional sensing sequences, and complementary relationships among sensing sources can be captured. Meanwhile, edge-computing systems impose higher requirements on model deployment. Models should process heterogeneous data from environmental, electrical, device, vibration, and network sources while maintaining robust prediction capability under load fluctuations, communication abnormalities, and device disturbances. In addition, industrial operation and maintenance scenarios and financial infrastructure monitoring scenarios focus not only on future continuous state values, such as service availability, response time, or task completion rate, but also on whether the current system is abnormal. Therefore, an effective industrial hardware sensing fusion framework should support cross-source signal alignment, disturbance-robust modeling, context-adaptive fusion, state prediction, and anomaly perception.

However, existing methods still have evident limitations in multisource hardware sensing fusion for industrial edge-computing systems. First, most methods lack an explicit cross-source alignment mechanism for asynchronous hardware sensing signals, making it difficult to learn time offsets and dynamic response relationships among sensing sources. Second, normal state evolution and abnormal disturbance responses are usually modeled within the same prediction branch, which may cause error accumulation in non-stationary disturbance windows. Third, fixed fusion weights or static attention structures cannot adapt well to changes in information contribution under different operational stages, sensor reliability states, and disturbance intensities. For example, communication-state signals may be more critical under network congestion, whereas environmental temperature, device temperature, and power-consumption signals may be more informative under restricted heat dissipation. Therefore, dynamically adjusting the contribution of different sensing sources according to system context and maintaining stable performance under partial sensor missingness or increased noise remain key challenges in industrial and financial–industrial state prediction and anomaly perception.

Based on the above issues, a multisource hardware sensing signal fusion network is proposed for robust state prediction and anomaly perception in complex industrial edge-computing, financial–industrial infrastructure, and digital production systems. Environmental sensing, equipment electrical states, edge-node operational states, vibration monitoring, network communication states, and system output states are uniformly modeled as multisource engineering sensing signals. An end-to-end prediction framework is then constructed to achieve joint optimization from heterogeneous sensing signal encoding, cross-source dynamic alignment, and disturbance residual correction to context-adaptive fusion. Different from traditional single-source prediction or simple feature-concatenation methods, the proposed method explicitly considers asynchronous response relationships and disturbance propagation mechanisms among hardware sensing sources. Through learnable time offsets, cross-source attention, gated residual correction, and reliability-constrained fusion, prediction stability and anomaly recognition capability are improved under complex non-stationary scenarios. The main contributions of this study are summarized as follows.

1. A multisource hardware sensing dataset is constructed for industrial edge-computing and digital production systems. The dataset includes environmental sensing, equipment power consumption, edge-node operational status, vibration, network communication, and system output states under normal and disturbed operating conditions.
2. A cross-source sensor signal alignment module is proposed to address the asynchrony of multisource hardware sensing data, thereby reducing temporal mismatches caused by simple feature concatenation.
3. A disturbance-aware residual correction module is designed to improve robustness under non-stationary disturbances. Stable trend modeling and short-term disturbance correction are separated through a base trend branch, a disturbance response branch, and a gated residual structure.
4. A context-adaptive multisource fusion module is proposed to dynamically integrate different sensing sources according to operational context, sensor validity, historical fluctuation, and disturbance intensity.

## 2. Related Work

### 2.1. Hardware Sensor-Based System State Monitoring and Prediction

Hardware sensor-based system state monitoring and prediction aims to continuously acquire and model multidimensional sensing signals generated during complex industrial and financial–industrial system operation, so that system evolution trends, operational risks, and potential anomalies can be perceived in advance [10]. Different hardware sensors can observe internal states and external disturbances from physical, environmental, communication, and operational layers, and the mapping between sensing signals and system states can be established through time-series modeling [11]. With the development of the Internet of Things, edge intelligence, and digital twin technologies, temperature and humidity sensors, vibration sensors, current-voltage sensors, cameras, RFID, GPS, network probes, and device monitoring modules have been widely deployed in intelligent manufacturing, smart agriculture, transportation systems, data centers, financial infrastructures, and digital infrastructures [12]. These sensors continuously record environmental variations, device states, network behaviors, and load fluctuations, forming sensing data streams for complex engineering systems [13]. Early studies mainly relied on single sensors or a few structured indicators for state prediction [14]. Vibration, temperature, or current signals are commonly used for equipment health prediction, whereas traffic, latency, and packet loss rate are used for network stability and financial service availability analysis [15]. These methods are often based on statistical modeling or rule-based analysis, providing strong interpretability and deployment convenience [16,17]. However, as engineering systems become larger and more coupled, single-sensor modeling is insufficient for characterizing multifactor interactions and nonlinear system dynamics.

Deep learning has improved sensor time-series modeling capability in industrial and finance-related digital systems [18]. LSTM and GRU capture long-term dependencies through gating mechanisms, TCN models multiscale temporal patterns through dilated convolutions, and Transformer establishes global dependencies through self-attention [19]. Graph neural networks further extend state prediction to spatiotemporal coupled modeling by learning device or node topology [20]. Nevertheless, stable, robust, and interpretable modeling remains challenging when multiple sensors, operational indicators, and disturbances coexist [21]. In complex digital systems, inconsistent sampling frequencies, different physical meanings, and delayed responses among sensors still hinder traditional single-sequence models [22]. Therefore, collaborative modeling of multisource hardware sensing has become an important research direction [23].

### 2.2. Multisource Sensor Fusion and Cross-Modal Signal Alignment

Multisource sensor fusion aims to learn unified representations from heterogeneous sensing signals collected from different devices, locations, and sampling mechanisms [24]. Based on multiview perception and information complementarity, different sensors reflect system states from different dimensions, while a single sensor usually captures only local information [25]. Therefore, multisource fusion can alleviate noise interference, missing information, and local bias in individual signals [26]. In engineering and financial–industrial systems, environmental sensors reflect external conditions, device sensors describe load and energy states, network signals measure transmission stability, and platform interaction or transaction-service data indirectly reflect operational outcomes [27,28]. Existing fusion methods include early fusion, late fusion, attention fusion, graph-structured fusion, and multimodal Transformer-based methods [29]. Early fusion directly concatenates features but struggles with heterogeneity and temporal-scale differences. Late fusion preserves modality independence but may ignore dynamic interactions [30]. Attention-based methods dynamically allocate weights to task-relevant sensing channels, while multimodal Transformer models establish deeper cross-modal associations through attention mechanisms [31,32].

However, strict synchronization rarely exists among multisource signals in complex digital systems [33]. Different sensors often show leading or lagging responses to the same state variation [34]. Environmental changes may first affect device load, device load may influence power consumption and latency, and network congestion may later affect platform outputs, including transaction-processing stability in financial–industrial infrastructures [35]. Simple time-window concatenation may therefore introduce spurious correlations and temporal misalignment noise [36]. Moreover, different sensors often have substantially different sampling frequencies, further increasing the difficulty of cross-source fusion [37]. Cross-source temporal alignment has therefore become important in multisource fusion [38]. Dynamic time warping, learnable time offsets, multiscale windows, and cross-modal attention are commonly used to establish dynamic correspondences and learn temporal response functions among sensors [39]. Learnable time-offset weights further allow models to capture leading, lagging, and collaborative relationships automatically [40]. However, existing studies mostly focus on specific scenarios or single-modality tasks, while dynamic propagation among environmental sensing, device operation, network communication, platform interaction, and finance-related service outputs remains insufficiently explored [41]. Therefore, a cross-source sensing signal alignment module is developed in this study to learn unified representations through cross-source attention and dynamic time-offset mechanisms [42].

### 2.3. Deep Learning for Disturbance-Aware Robust Prediction

Complex engineering and financial–industrial systems are often affected by environmental changes, device failures, network anomalies, load shocks, transaction-service surges, and human strategy adjustments, leading to short-term transitions, distribution shifts, and non-stationary variations [43]. Disturbance-aware prediction aims to improve the model response to abrupt events while preserving long-term trend modeling capability [44]. Traditional time-series models usually assume stable data distributions and rely on historical regularity, making them prone to prediction lag and error accumulation under abrupt disturbances [45,46]. Deep learning has been widely used for robust prediction [47]. Event-aware modeling encodes alarms or external disturbances as auxiliary inputs, gating mechanisms separate normal trends from abnormal shocks, and residual correction structures compensate for abrupt state changes by learning base-trend errors [48]. Attention mechanisms and uncertainty modeling further enhance robustness by assigning dynamic weights across time windows and sensing channels and estimating prediction confidence under abnormal conditions [49].

Although progress has been made, most methods focus on a single event type or scenario, such as device fault prediction, network anomaly detection, financial service anomaly perception, or load fluctuation analysis. Differences in sensor responses to disturbance events remain insufficiently studied from the perspective of multisource hardware sensing fusion [50]. In complex digital systems, environmental disturbances may first affect environmental sensors and then propagate to device and network states, whereas platform activity shocks may first appear as traffic anomalies and later affect system outputs [51]. Therefore, a single disturbance modeling strategy is insufficient for describing complex state transition processes.

## 3. Materials and Method

### 3.1. Data Collection

The dataset used in this study was collected from an edge-computing and digital production operation test platform of an intelligent equipment manufacturing enterprise located in Shijiazhuang, Hebei Province, China, which provides an infrastructure-level scenario relevant to industrial and financial–industrial monitoring. The data acquisition sites mainly included edge nodes in the production workshop, equipment control cabinets, network switch rooms, and a small-scale digital system test area, as shown in Table 1, Table 2 and Table 3. Data collection was jointly organized by the enterprise technical department and the research team under a unified experimental protocol. The collection period lasted from May 2024 to October 2024, covering multiple complex system states, including normal equipment operation, production workload fluctuations, network communication congestion, variations in cabinet temperature and humidity, high-power operation of edge nodes, equipment vibration disturbances, and short-term abnormal events.

Environmental sensing data were mainly obtained from Sensirion SHT31 temperature and humidity sensors, BH1750 illumination sensors, and Bosch BMP280 barometric pressure sensors deployed around edge-computing nodes in the production workshop, inside equipment control cabinets, and in network switch rooms. These sensors were used to record temperature, relative humidity, illumination intensity, and barometric pressure, with a sampling interval of 1 min. Equipment power data were collected using INA219 voltage–current sensors, which were used to acquire voltage, current, and power-consumption variations in real time, with a sampling period of 5 s. Edge-device operational status data were jointly collected from NVIDIA Jetson Xavier NX edge-computing nodes and Raspberry Pi 4B data acquisition terminals. The Jetson Xavier NX nodes were used to support edge inference, status monitoring, and local computing tasks, while the Raspberry Pi 4B devices served as sensor gateways for aggregating multisource hardware signals. The recorded device-status variables included CPU load, GPU load, memory usage, and device temperature.

Equipment vibration data were collected using ADXL345 three-axis accelerometers, which were fixed near the housings of edge-computing devices, the support structures of control cabinets, and cooling fans. These sensors were used to capture X-axis acceleration, Y-axis acceleration, Z-axis acceleration, vibration amplitude, mechanical vibration, fan-induced disturbances, and local impact signals during equipment operation, with a raw sampling frequency of 50 Hz. Network communication status data were obtained from TP-Link TL-SG108E managed switch port statistics and Raspberry Pi network probes. The recorded variables mainly included uplink traffic, downlink traffic, request frequency, average response latency, packet loss rate, and the number of abnormal connections, with a sampling interval of 10 s. To obtain representative disturbance samples, controllable experimental conditions, including increased edge-computing workload, bandwidth limitation, short-term network disconnection, restricted heat dissipation, and environmental temperature–humidity fluctuations, were introduced during data collection under the premise of not affecting normal production safety. The start time, duration, impact range, and recovery process of each disturbance were recorded using programmable workload control modules, network rate-limiting and disconnection control modules, hardware alarm modules, and system monitoring programs.

System output states were automatically recorded by the enterprise edge monitoring program, including task completion rate, average response time, service availability score, and abnormal state labels. To ensure auditability and construct a highly reliable ground truth, a rigorous manual verification and cross-validation protocol was introduced. Specifically, three independent domain experts participated in the labeling process. A synchronized time window was provisionally labeled as abnormal if it met any of the following strict threshold criteria: the average response time exceeded 2000 ms, the CPU load remained above 95% for three consecutive minutes alongside a hardware thermal alarm, or the network packet loss rate exceeded 5%. Following this automated preliminary screening, the three annotators independently reviewed the corresponding equipment logs and sensor trajectories. The final ground-truth label was determined through a majority voting mechanism among the experts. To quantify the consistency among the annotators, the Cohen kappa coefficient was calculated, achieving an average inter-annotator agreement score of 0.89, which quantitatively indicates a highly consistent and reliable validation procedure. All hardware sensing nodes were connected to a unified time synchronization server, and timestamps were written at the acquisition end to ensure that environmental, electrical, equipment-status, vibration, network-communication, disturbance-event, and system-output data could be aligned within a unified time window for subsequent modeling.

After aligning the heterogeneous time-series data using a unified time window of 1 min, we obtained a total of 263,520 synchronized samples. It should be noted that these samples are not derived from a single continuous timeline but consist of aggregated concurrent windows collected simultaneously from multiple active edge nodes, which accounts for the total sample volume exceeding the strict chronological minute count of the collection period. The overall missing-data rate prior to imputation was 4.2%, primarily caused by temporary sensor disconnection and network jitter. Based on the established labeling criteria, the class balance of the synchronized dataset consists of 88.5% normal operation samples and 11.5% abnormal samples. Furthermore, the dataset contains exactly 120 controlled disturbance events, including 30 edge-computing load surges, 30 bandwidth limitations, 20 short-term network disconnections, 20 restricted heat dissipation events, and 20 environmental fluctuations. The exact distribution of the normal and abnormal classes, their corresponding disturbance types, and the number of samples per category are detailed in Table 3. To prevent temporal leakage during evaluation, the dataset was chronologically partitioned, with the training set spanning from 1 May 2024 to 15 August 2024, the validation set from 16 August 2024 to 7 September 2024, and the test set from 8 September 2024 to 15 October 2024.

### 3.2. Data Augmentation

In complex digital systems, multisource hardware sensors continuously generate heterogeneous sensing signals with different sampling mechanisms, temporal granularities, and data formats. Therefore, the core objective of data preprocessing and augmentation is to map data from different sensors into a unified state modeling space through unified temporal representation, standardized feature spaces, and enhanced disturbance robustness, thereby providing stable inputs for subsequent cross-source alignment and multimodal fusion. Since environmental sensors, device operation monitoring modules, network communication probes, and platform interaction logs differ significantly in physical meaning, sampling frequency, and statistical distribution, direct input of raw data into the prediction model may easily lead to temporal misalignment, scale bias, and noise accumulation. Therefore, multisource sensing signals need to be uniformly processed from multiple aspects, including temporal synchronization, feature normalization, semantic encoding, and disturbance augmentation.

First, unified time-window alignment is required for multisource sensing signals. The basic principle is to map heterogeneous time-series signals with different sampling frequencies onto a unified temporal scale so that cross-source state associations can be established. Suppose that there are *M* types of sensors in the system, and the raw observation value of the *m*-th sensor at time *t* is denoted as $x_{m}\left(t\right)$. Since different sensors have different sampling intervals $\Delta t_{m}$, a unified time window $W_{k}=\left[t_{k},t_{k}+\Delta T\right]$ is constructed, and the raw signals are aggregated within the unified window. For high-frequency sensor signals, statistical aggregation is adopted to extract local dynamic features, including the mean, variance, extreme values, and change rate. The calculation is formulated as follows:

(1) $$\mu_{m}^{\left(k\right)}=\frac{1}{|W_{k}|}\sum_{t\in W_{k}}x_{m}\left(t\right),$$

(2) $$\sigma_{m}^{\left(k\right)}=\sqrt{\frac{1}{|W_{k}|}\sum_{t\in W_{k}}{\left(x_{m}\left(t\right)-\mu_{m}^{\left(k\right)}\right)}^{2}},$$

(3) $$r_{m}^{\left(k\right)}=\frac{x_{m}\left(t_{k}+\Delta T\right)-x_{m}\left(t_{k}\right)}{\Delta T},$$

where $\mu_{m}^{\left(k\right)}$ denotes the mean feature of the *m*-th sensor within the window $W_{k}$, $\sigma_{m}^{\left(k\right)}$ denotes the local fluctuation intensity, and $r_{m}^{\left(k\right)}$ is used to describe the variation trend within the window. For low-frequency state variables, missing values may occur within a window due to their low update frequency; therefore, a combination of forward filling and interpolation is adopted. Let the missing position be $t_{i}$, and let the interpolation result be expressed as follows:

(4) $$\hat{x}\left(t_{i}\right)=x\left(t_{i-1}\right)+\frac{t_{i}-t_{i-1}}{t_{i+1}-t_{i-1}}\left(x\left(t_{i+1}\right)-x\left(t_{i-1}\right)\right).$$

In addition, event-type signals, such as abnormal alarms, state transitions, and access surges, are further encoded into structured representations, including event intensity, duration, and occurrence frequency. Let the event set be denoted as E. The event intensity within the time window $W_{k}$ can be defined as follows:

(5) $$E_{k}=\sum_{e_{i}\in E_{k}}w_{i}\cdot d_{i},$$

where $E_{k}$ denotes the set of disturbance events within the *k*-th time window, $E_{k}$ denotes the calculated overall event intensity, $w_{i}$ denotes the event weight, and $d_{i}$ denotes the event duration. Through the above processing, sensing data from different sources are uniformly mapped into state representations under a fixed temporal granularity, thereby alleviating temporal misalignment caused by heterogeneous sampling frequencies. After temporal alignment is completed, numerical sensing variables need to be further standardized. The theoretical basis is that the numerical ranges and statistical distributions of different sensor outputs usually differ significantly. For example, access traffic and power-consumption data usually exhibit long-tailed distributions, whereas continuous variables such as temperature, voltage, and latency show relatively stable Gaussian-like distributions. Without normalization, large-scale variables may dominate gradient updates during model training, thereby reducing the sensitivity of the model to other sensing channels. Therefore, differentiated standardization strategies are adopted for different variable types. For long-tailed variables such as access frequency, network traffic, and system output states, logarithmic transformation is adopted to reduce the influence of extreme values, as formulated below:

(6) $$x_{i}^{\prime }=\log\left(x_{i}+\epsilon \right),$$

where ϵ is a smoothing term used to prevent numerical overflow. For continuous variables such as temperature, humidity, voltage, current, and load, *z*-score standardization is adopted:

(7) $$z_{i}=\frac{x_{i}-\mu }{\sigma },$$

where μ and σ denote the mean and standard deviation of the variable, respectively. In addition, for certain variables with fixed upper and lower bounds, Min–Max normalization can also be applied:

(8) $$x_{i}^{norm}=\frac{x_{i}-x_{min}}{x_{max}-x_{min}}.$$

For discrete variables such as device type, status code, object category, and event label, an embedding mapping is further adopted to obtain dense representations. Let the discrete variable be $c_{i}$, and let the embedding matrix be $E\in R^{V\times d}$. The corresponding vector representation is given by the following:

(9) $$h_{i}=E\left(c_{i}\right),$$

where *V* denotes the total number of categories, and *d* denotes the embedding dimension. Through embedding mapping, discrete state variables can be transformed into low-dimensional representations in a continuous semantic space, thereby enhancing the model’s ability to learn category associations. In addition to structured numerical data, complex digital systems also contain a large amount of platform interaction text and log text, such as user feedback, abnormal alarm descriptions, system logs, and operation records. Although these text data do not constitute the core background of this study, important semantic cues contained in them can be used as auxiliary observation modalities for system state modeling. Therefore, unified cleaning and semantic encoding need to be performed on textual data. First, noise interference is reduced by removing duplicate texts, abnormally short texts, invalid characters, and stop symbols. Then, a pretrained language model is used to extract textual semantic representations. Let the text sequence be $T=w_{1},w_{2},\ldots ,w_{n}$. The semantic representation output by the text encoder $f_{\theta }\left(\cdot \right)$ is given by the following:

(10) $$h_{T}=f_{\theta }\left(w_{1},w_{2},\ldots ,w_{n}\right).$$

Furthermore, to enhance the model’s perception of contextual semantic relationships, the Transformer self-attention mechanism can be used for text representation learning. Its attention calculation is formulated as follows:

(11) $$Attention\left(Q,K,V\right)=Softmax\left(\frac{QK^{T}}{\sqrt{d_{k}}}\right)V,$$

where *Q*, *K*, and *V* denote the query matrix, key matrix, and value matrix, respectively, and $d_{k}$ denotes the feature dimension. Through this mechanism, key semantic dependencies in text sequences can be automatically learned, and the textual modality can be jointly mapped with the hardware sensor modality into a unified feature space. To further improve the generalization capability and robustness of the model under complex scenarios, data augmentation strategies for multisource sensing signals are also designed. The core idea is to simulate unstable states in real deployment environments by artificially constructing noise, missingness, and temporal perturbations, thereby enhancing the adaptability of the model to abnormal conditions. For time-series sensing data, sliding windows and random masking strategies are adopted to generate samples with different temporal contexts. Let the original sequence be $X=x_{1},x_{2},\ldots ,x_{T}$. The randomly masked sequence is represented as follows:

(12) $${\tilde{x}}_{t}=m_{t}\cdot x_{t},$$

where $m_{t}\in \left\{0,1\right\}$ denotes a random mask variable. In addition, to simulate sensing noise in practical environments, Gaussian perturbations can also be added to the original signals:

(13) $${\tilde{x}}_{t}=x_{t}+N\left(0,\sigma^{2}\right).$$

To enhance the adaptability of the model to sampling offsets and temporal-scale changes, local segment resampling and temporal-scale perturbation strategies are further adopted. Let the original time series be X(t). The perturbed temporal mapping function is expressed as follows:

(14) $$\tilde{X}\left(t\right)=X\left(\alpha t+\beta \right),$$

where α denotes the temporal scaling factor, and β denotes the time offset. For disturbance event data, local window samples before, during, and after the disturbance are further constructed, so that the model can learn the dynamic evolution process of system states from stability to abnormality and then to recovery. In addition, sensor failure, communication interruption, and data delay are common in real deployment scenarios. Therefore, a random channel masking mechanism is further adopted to simulate multisource missing scenarios. Let the fused input be $H=[h_{1},h_{2},\ldots ,h_{M}]$. The input after random missingness is represented as follows:

(15) $$\tilde{H}=\left[m_{1}h_{1},m_{2}h_{2},\ldots ,m_{M}h_{M}\right],$$

where $m_{i}\in \left\{0,\;1\right\}$ indicates whether the *i*-th sensor channel is available. In this way, the model can learn state reasoning capability under partial sensor failure conditions during training, thereby improving practical deployment robustness in complex digital systems.

### 3.3. Problem Formulation

Before detailing the methodology and evaluation metrics, it is essential to formally define the mathematical target variables for the state prediction and anomaly perception tasks. The system modeling in this study is decoupled into a continuous regression task and a discrete classification task. For the regression task, the model is required to predict the future state of the system over a prediction horizon of 7 time steps. The mathematical target variable *y* for this regression task is strictly defined as the service availability score of the edge-computing system. For the anomaly perception task, the problem is formulated as a binary classification task with a strict temporal offset to prevent target leakage. Specifically, the model utilizes historical multisource data up to time *t* to predict whether the system will experience an anomalous event within the future prediction horizon from time t+1 to t+7. The target variable *y* in this context is a binary label indicating this future state, where the value 0 represents a normal operational state and the value 1 represents a future abnormal state triggered by hardware alarms or network threshold violations.

### 3.4. Proposed Method

#### 3.4.1. Overall

The proposed multisource hardware sensing signal fusion network takes the processed multisource engineering sensing sequences as input and is constructed following the pipeline of source-specific encoding, cross-source alignment, disturbance correction, adaptive fusion, and state prediction. First, environmental sensing signals, device operational signals, network communication signals, platform interaction signals, and historical system states are fed into their corresponding encoding branches. Temporal encoders, structured-state encoders, event encoders, and log-semantic encoders are used to extract the hidden representations of different signal sources, thereby preserving their differences in temporal dynamics, operational attributes, and event semantics. Subsequently, the outputs of these branches are fed into the cross-source sensor signal alignment module, where the association strength among different signals is computed through a cross-source attention mechanism. Learnable time-offset weights are further incorporated to characterize the leading, lagging, and collaborative response relationships among sensing signals, so that asynchronously varying multisource signals can be mapped into a unified predictive representation space. On this basis, the disturbance-aware residual correction module further receives the aligned system-state representation and the event-disturbance representation. A base prediction branch is first used to learn stable operational trends, while a disturbance branch generates residual correction terms according to load surges, abnormal alarms, network peaks, or external shocks. A gating mechanism is then used to control the disturbance intensity, thereby preventing short-term anomalies from being incorrectly learned as long-term trends. Afterward, the context-adaptive fusion module uses object attributes, operational stages, sensor reliability, and disturbance intensity as contextual information to dynamically generate fusion weights for different signal sources. Environmental, device, network, interaction, and historical-state representations are then integrated through weighted aggregation. Finally, the fused system-state representation is fed into the prediction head to output future state values, demand levels, risk grades, or anomaly probabilities. The overall framework enables end-to-end modeling from heterogeneous sensing signal representation to cross-source dynamic association learning, disturbance-robust correction, and context-adaptive prediction.

#### 3.4.2. Cross-Source Sensor Signal Alignment Module

**Figure 1:**
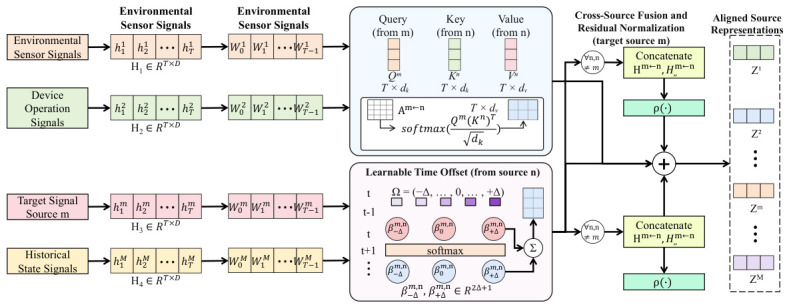
Structure of the Cross-Source Sensor Signal Alignment Module, which learns cross-source attention and learnable time-offset relationships to generate aligned multisource representations.

The core objective of the Cross-Source Sensor Signal Alignment Module is to establish dynamic response relationships among heterogeneous sensing signals under a unified prediction-task constraint. As shown in Figure 1, the representation of the *m*-th sensing signal obtained from the preceding encoder is denoted as $H^{m}=\left\{h_{1}^{m},h_{2}^{m},\ldots ,h_{T}^{m}\right\}$, where m∈{1,2,…,M} denotes the signal-source type, *T* denotes the length of the historical time window, and $h_{t}^{m}\in R^{d}$ represents the hidden state of the *m*-th signal at time window *t*. Since environmental sensing, device operation, network communication, platform interaction, and historical-state signals have different sampling mechanisms and response speeds, direct concatenation at the same time point implicitly assumes that all signals vary synchronously, which is inconsistent with the actual propagation process in complex digital systems. Therefore, an independent linear projection layer is first assigned to each sensing source to map it into a shared alignment space, namely, $Q^{m}=H^{m}W_{Q}^{m}$, $K^{m}=H^{m}W_{K}^{m}$, and $V^{m}=H^{m}W_{V}^{m}$, where $W_{Q}^{m}$, $W_{K}^{m}$, and $W_{V}^{m}$ are learnable parameters. Then, the query representation $Q^{m}$ of the target signal source *m* and the key representation $K^{n}$ of the reference signal source *n* are used to calculate the cross-source attention score, thereby characterizing the correlation between two types of signals at different temporal positions. This process is formulated as $A^{m,n}=\mathrm{softmax}\left(Q^{m}{\left(K^{n}\right)}^{T}/\sqrt{d}\right)$, and the response representation of the reference source to the target source is obtained as ${\tilde{H}}^{m,n}=A^{m,n}V^{n}$.

To further model leading and lagging effects, learnable time-offset weights are introduced in addition to the cross-source attention mechanism. Specifically, for the target time step *t*, the model does not only use the state of the reference source at the same time step, but it also aggregates candidate responses from the reference source within a local offset set Ω, which is given by ${\bar{h}}_{t}^{m,n}=\sum_{\delta \in \Omega }\beta_{\delta }^{m,n}h_{t+\delta }^{n}$. Here, $\beta_{\delta }^{m,n}=\mathrm{softmax}\left(e_{\delta }^{m,n}\right)$ denotes the response weight of source *n* relative to source *m* under the time offset δ, and $e_{\delta }^{m,n}$ is a learnable offset score. This design enables the model to automatically determine whether a specific sensing signal leads, synchronizes with, or lags behind another signal, without the need to manually specify a fixed time delay. Subsequently, the cross-source attention representation and the time-offset representation are further fused to form the aligned target-source representation $Z^{m}=\mathrm{LayerNorm}\left(H^{m}+\sum_{n\neq m}\gamma^{m,n}\phi \left(\left[{\tilde{H}}^{m,n};{\bar{H}}^{m,n}\right]\right)\right)$, where $\gamma^{m,n}$ denotes the cross-source gating weight, ϕ(·) denotes a nonlinear mapping function, and [;] denotes feature concatenation. This residual normalization structure preserves the intrinsic dynamic features of the target source while incorporating information from other signal sources, thereby preventing the original sensing features from being diluted by excessive cross-source fusion. Finally, all aligned source representations $\left\{Z^{1},Z^{2},\ldots ,Z^{M}\right\}$ are fed into the subsequent disturbance-aware residual correction module and context-adaptive multisource fusion module.

To fundamentally distinguish this approach from a conventional assembly of existing attention mechanisms, it is essential to emphasize its specific algorithmic suitability for industrial edge-computing environments. In actual edge digital systems, heterogeneous sensors operate at intrinsically different sampling frequencies, such as mechanical vibration sensors running at 50 Hz versus environmental temperature sensors updating at 1 min intervals, and physical processes exhibit inherent transmission delays, such as thermal accumulation leading to a subsequent CPU throttling event. The synergistic combination of cross-source attention and learnable time offsets elegantly addresses these exact industrial pain points. Rather than forcing asynchronous signals into an artificial synchronous matrix, the learnable offsets act as a temporal buffer that aligns the physical causality of events across variable sampling rates, while the cross-source attention dynamically filters out the resulting frequency-mismatch noise. This specific structural design guarantees that the subsequent prediction networks process a temporally and causally coherent representation, strictly preventing the asynchronous propagation delays of hardware sensors from collapsing the predictive stability.

#### 3.4.3. Disturbance-Aware Residual Correction Module

The Disturbance-Aware Residual Correction Module is designed to separately model stable evolutionary trends and abrupt disturbance responses in complex digital systems, thereby avoiding significant prediction deviations under load surges, network congestion, sensor anomalies, or external event shocks.

**Figure 2:**
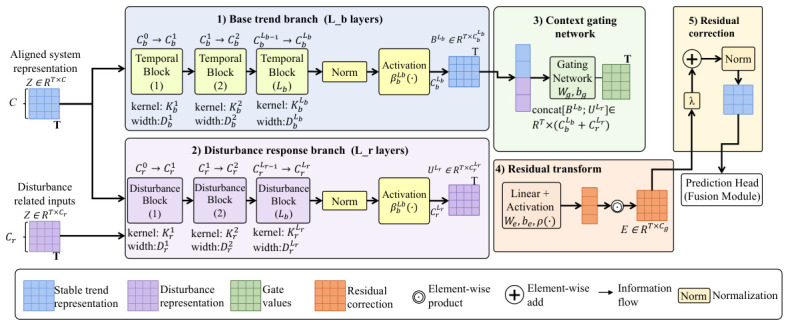
Structure of the Disturbance-Aware Residual Correction Module, which separates stable trend modeling from disturbance response modeling through a gated residual correction mechanism.

As shown in Figure 2, the system-state representation output by the cross-source alignment module is denoted as $Z\in R^{T\times C}$, where *T* denotes the time-window length and *C* denotes the aligned channel dimension. The disturbance-related input is denoted as $R\in R^{T\times C_{r}}$, which consists of event identifiers, abnormal alarms, load-change rates, network peaks, sensor fluctuation amplitudes, and external disturbance intensities. This module adopts a dual-branch architecture. The base trend branch consists of $L_{b}$ layers of one-dimensional temporal convolutional blocks or feed-forward temporal blocks, where the input channel number, output channel number, and hidden width of each layer are denoted as $C_{b}^{l-1}$, $C_{b}^{l}$, and $D_{b}^{l}$, respectively, and the kernel width is denoted as $K_{b}^{l}$. This branch is used to extract stable state-evolution features. The disturbance response branch consists of $L_{r}$ layers of disturbance encoding blocks, where the channel number and hidden width of each layer are denoted as $C_{r}^{l}$ and $D_{r}^{l}$, respectively. A gating unit is further incorporated to estimate the effective disturbance intensity at the current time step. The base branch does not directly output the final prediction but generates a stable-state embedding *B*, while the disturbance branch generates a disturbance-response embedding *U*. These two embeddings are dynamically coupled through a gated residual structure. Specifically, the *l*-th layer of base trend extraction is formulated as follows:

(16) $$B^{l}=\psi_{b}^{l}\left(\mathrm{Norm}\left(F_{b}^{l}\left(B^{l-1};\Theta_{b}^{l}\right)\right)\right),\;B^{0}=Z,$$

where $F_{b}^{l}\left(\cdot \right)$ denotes the temporal mapping function of the *l*-th layer, $\Theta_{b}^{l}\in R^{K_{b}^{l}\times C_{b}^{l-1}\times C_{b}^{l}}$ denotes its parameter tensor, and $\psi_{b}^{l}\left(\cdot \right)$ denotes the nonlinear activation function. The disturbance response branch first maps the disturbance input into a hidden space compatible with the base trend representation:

(17) $$U^{l}=\psi_{r}^{l}\left(\mathrm{Norm}\left(F_{r}^{l}\left(U^{l-1};\Theta_{r}^{l}\right)\right)\right),\;U^{0}=R,$$

where $\Theta_{r}^{l}\in R^{K_{r}^{l}\times C_{r}^{l-1}\times C_{r}^{l}}$ denotes the disturbance encoding parameters. To control the influence of disturbance terms on the prediction result, a contextual gating function is further designed by feeding both the stable trend embedding and the disturbance embedding into the gating network:

(18) $$G=\sigma \left(W_{g}\left([B^{L_{b}};U^{L_{r}}]\right)+b_{g}\right),$$

where $B^{L_{b}}\in R^{T\times C}$ denotes the base trend tensor, $U^{L_{r}}\in R^{T\times C_{r}}$ denotes the disturbance response tensor, and $G\in {\left[0,1\right]}^{T\times C}$ denotes the calculated disturbance gating matrix. To maintain dimensional compatibility, the input width of $W_{g}$ is $C+C_{r}$, and the output channel number is strictly set to *C*. Subsequently, the disturbance response is not directly added to the system representation but is first transformed into a controlled correction term through a residual transformation function:

(19) $$E=G⊙\rho \left(W_{e}U^{L_{r}}+b_{e}\right),$$

where ⊙ denotes element-wise multiplication, ρ(·) denotes a nonlinear mapping, and $E\in R^{T\times C}$ denotes the local state offset caused by the disturbance. Here, the linear projection $W_{e}$ maps the original disturbance representation $U^{L_{r}}$ exactly to the dimension of $R^{T\times C}$, guaranteeing that it perfectly aligns with the gating matrix *G* for the element-wise multiplication operation. Finally, the disturbance-corrected representation output by the module is given by the following:

(20) $$Z_{c}=\mathrm{Norm}\left(B^{L_{b}}+\lambda E\right),$$

where λ is a learnable scaling factor used to balance the relative contributions of stable trends and disturbance responses. Because both the base trend tensor $B^{L_{b}}$ and the local state offset tensor *E* are strictly mapped to the identical dimension of $R^{T\times C}$, the element-wise residual addition is mathematically valid and seamlessly produces the final representation $Z_{c}\in R^{T\times C}$. To demonstrate the rationality of this design, the true future state representation can be decomposed into a stable term and a disturbance term, namely, Y=S+ϵ, where *S* denotes the predictable component determined by the long-term trend, and ϵ denotes the short-term deviation caused by abrupt disturbances. If a single model is used to directly fit *Y*, its expected risk can be expressed as follows:

(21) $$R_{single}=E\left[{∥Y-f(Z,R)∥}_{2}^{2}\right].$$

When the statistical patterns of the disturbance term ϵ and the stable term *S* are inconsistent, a single mapping is prone to parameter conflicts between stable samples and disturbed samples. To provide an intuitive theoretical motivation rather than a formal mathematical proof, we assume ideal conditions where the residual term ϵ satisfies a zero-mean distribution and the gating function can converge to the optimal conditional expectation based on the disturbance context. Under these assumptions, the predictive representation is decomposed into a trend representation and a gated disturbance representation, which is equivalent to learning the main mapping of *S* and the conditional correction mapping of ϵ. The corresponding risk can be written as follows:

(22) $$R_{dec}=E\left[{∥S-f_{s}\left(Z\right)∥}_{2}^{2}\right]+E\left[{∥\epsilon -G⊙f_{r}\left(R\right)∥}_{2}^{2}\right].$$

Assuming that the residual term ϵ satisfies a zero-mean distribution and that the gating function can converge to the optimal conditional expectation based on the disturbance context, $G⊙f_{r}\left(R\right)$ becomes a closer estimator of E[ϵ|R,Z], thereby reducing the residual error in disturbance windows:

(23) $$E\left[{∥\epsilon -G⊙f_{r}\left(R\right)∥}_{2}^{2}\right]\leq E\left[{∥\epsilon -f_{r}\left(R\right)∥}_{2}^{2}\right].$$

It should be noted that the inequality derivations presented above serve as an intuitive theoretical motivation rather than a strict mathematical proof. Based on this theoretical intuition, the algorithmic design of separating the stable trend from short-term deviations provides a critical advantage tailored for industrial edge environments characterized by non-stationary, high-frequency physical disturbances. For example, when an abrupt mechanical vibration spike or a transient network drop occurs, a conventional unified model would inevitably adjust its global weights, thereby polluting the long-term baseline features with localized noise. By confining the disturbance fitting entirely within the residual correction branch, the base temporal network is structurally shielded from these high-frequency shocks. As a result, the model strictly prevents short-term anomalies from being incorrectly absorbed as long-term evolutionary patterns, fundamentally improving the robustness, event-window adaptability, and cross-scenario generalization capability of state prediction in complex digital systems.

#### 3.4.4. Context-Adaptive Multisource Fusion Module

The Context-Adaptive Multisource Fusion Module is used to dynamically integrate multisource representations under different system objects, operational stages, sensor reliability conditions, and disturbance contexts, so that the model no longer relies on a fixed fusion strategy.

**Figure 3:**
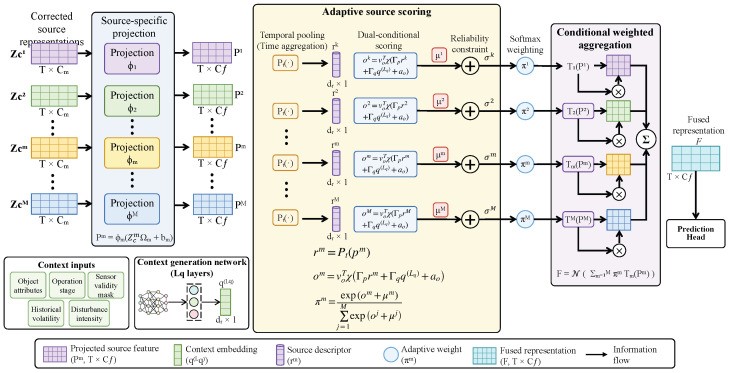
Structure of the Context-Adaptive Multisource Fusion Module, which dynamically assigns source-specific fusion weights according to contextual states and sensor reliability.

As shown in Figure 3, the multisource representations after cross-source alignment and disturbance correction are denoted as $Z=Z_{c}^{1},Z_{c}^{2},\ldots ,Z_{c}^{M}$, where *M* denotes the number of signal sources, $Z_{c}^{m}\in R^{T\times C_{m}}$ denotes the corrected state representation of the *m*-th signal source, *T* denotes the temporal dimension, and $C_{m}$ denotes the channel number of this source. Since the channel dimensions of different signal sources may be inconsistent, source-specific projection layers are first adopted to map all modalities into a unified fusion space. The input channel number and output channel number of each projection layer are $C_{m}$ and $C_{f}$, respectively, formulated as follows:

(24) $$P^{m}=\varphi_{m}\left(Z_{c}^{m}\Omega_{m}+b_{m}\right),\;\Omega_{m}\in R^{C_{m}\times C_{f}},$$

where $P^{m}\in R^{T\times C_{f}}$ denotes the unified representation of the *m*-th signal source, and $\varphi_{m}\left(\cdot \right)$ denotes the nonlinear mapping function. Subsequently, a context generation network is constructed to encode object attributes, operational stages, sensor validity masks, historical fluctuation statistics, and disturbance intensity into a context vector $q\in R^{C_{q}}$. This context network consists of $L_{q}$ layers of feed-forward mappings. The input width and output width of the *l*-th layer are denoted as $D_{q}^{l-1}$ and $D_{q}^{l}$, respectively, and the corresponding parameter matrix is $\Pi_{q}^{l}\in R^{D_{q}^{l-1}\times D_{q}^{l}}$. The computation process is given by the following:

(25) $$q^{l}=\eta_{q}^{l}\left(q^{l-1}\Pi_{q}^{l}+a_{q}^{l}\right),\;q^{0}=q.$$

To make the fusion weights simultaneously depend on contextual states and intra-source feature distributions, static weights are not directly assigned to each source. Instead, a dual-conditioned scoring function is used to calculate the adaptive response of each source. Specifically, the *m*-th signal source is first processed by a temporal compression function to obtain a global source descriptor $r^{m}$, and then the source reliability score is generated jointly with the context embedding:

(26) $$r^{m}=P_{t}\left(P^{m}\right),\;o^{m}=v_{o}^{T}\chi \left(\Gamma_{p}r^{m}+\Gamma_{q}q^{L_{q}}+a_{o}\right),$$

where $P_{t}\left(\cdot \right)$ denotes the temporal aggregation function, $r^{m}\in R^{C_{f}}$, $\Gamma_{p}\in R^{C_{f}\times D_{o}}$ and $\Gamma_{q}\in R^{D_{q}^{L_{q}}\times D_{o}}$ are conditional scoring mapping parameters, $D_{o}$ denotes the hidden width of the scoring network, χ(·) denotes the activation function, and $v_{o}$ is the output projection vector. To prevent a specific signal source from being over-amplified when it is noisy or severely missing, a reliability constraint term $\mu^{m}$ is further introduced. This term is jointly determined by sensor validity, missing ratio, and local fluctuation stability, and it is used to revise the source score:

(27) $$\pi^{m}=\frac{\exp(o^{m}+\mu^{m})}{\sum_{j=1}^{M}\exp\left(o^{j}+\mu^{j}\right)}.$$

Here, $\pi^{m}$ denotes the adaptive contribution coefficient of the *m*-th signal source under the current context. Finally, the multisource fusion representation is obtained through conditional weighted aggregation:

(28) $$F=N\left(\sum_{m=1}^{M}\pi^{m}T_{m}\left(P^{m}\right)\right),$$

where $T_{m}\left(\cdot \right)$ denotes the source-specific transformation function, N(·) denotes the normalization operation, and $F\in R^{T\times C_{f}}$ represents the final state representation input into the prediction head. From a mathematical perspective, the theoretical advantage of this module can be understood as an intuitive motivation under a context-conditioned risk minimization framework. Assuming ideal conditions where the error distributions of individual sensors are independent given the context, let F* denote the optimal fused representation, which consists of conditionally effective information from different sources. A fixed fusion strategy can only learn a set of context-independent coefficients, whereas the proposed conditional fusion strategy approximates the posterior contribution of each source under the current state through $\pi^{m}=p\left(m|q,r^{m}\right)$. Under the assumption that the scoring network optimally captures source relevance, if a certain source has lower noise and higher predictive relevance under the context *q*, its conditional error satisfies $E^{m}\left(q\right)<E^{j}\left(q\right)$, and ideally $\pi^{m}>\pi^{j}$ should hold. Therefore, the conditional expected error of dynamic fusion can be written as follows:

(29) $$E_{ada}=E_{q}\left[\sum_{m=1}^{M}\pi^{m}\left(q\right)E^{m}\left(q\right)\right],$$

whereas the error of fixed fusion is expressed as follows:

(30) $$E_{fix}=E_{q}\left[\sum_{m=1}^{M}{\bar{\pi }}^{m}E^{m}\left(q\right)\right].$$

When the distribution of optimal signal sources varies across different contexts, there exists $\pi^{m}\left(q\right)$ that can better approximate the optimal conditional weights than the fixed coefficient ${\bar{\pi }}^{m}$, and therefore, the following holds:

(31) $$E_{ada}\leq E_{fix}.$$

This derivation indicates that context-adaptive fusion can theoretically reduce the scene-mismatch risk caused by fixed fusion. When applied to the task in this study, this module can automatically adjust signal contributions under different states, such as environment-sensitive, load-sensitive, network-sensitive, or interaction-sensitive conditions. A more stable system-state representation can therefore be maintained under sensor missingness, noise enhancement, and disturbance shocks. Meanwhile, $\pi^{m}$ can also be used as an interpretable measure of sensing-source contribution, allowing the key signal sources relied upon by the model at different operational stages to be analyzed and thereby enhancing the interpretability of state prediction in complex digital systems.

## 4. Results and Discussion

### 4.1. Experimental Configuration

#### 4.1.1. Hardware and Software Platform

In terms of the hardware environment, the experiments in this study were conducted on a high-performance deep learning server platform. The server was equipped with an Intel Xeon Gold series multi-core CPU with a clock frequency of 2.4 GHz and 256 GB DDR4 memory to meet the requirements of large-scale multisource sensing data loading and parallel computation. For GPU acceleration, a hybrid computing platform consisting of NVIDIA RTX 4090 and NVIDIA A100 GPUs was adopted, with single-card memory capacities of 24 GB and 80 GB, respectively. This configuration was used to support the training and inference of Transformer models, multimodal fusion networks, and large-scale time-series models. Data storage was implemented using NVMe SSD high-speed solid-state drives to improve the reading efficiency of multisource time-series data and log files. The overall hardware platform was sufficient to meet the computational requirements of multimodal feature encoding, cross-source temporal alignment, and disturbance-aware modeling in the complex digital system state prediction task.

In terms of the software environment, the experiments were implemented on the Ubuntu 22.04 LTS operating system. PyTorch 2.1 was used as the deep learning framework, with CUDA 12.1 and cuDNN 8.9 adopted for GPU acceleration. Data preprocessing and statistical analysis were mainly implemented using NumPy, Pandas, and Scikit-learn. Time-series modeling and deep neural network training were completed based on PyTorch Lightning. Some text semantic encoding modules were implemented using HuggingFace Transformers for pretrained model loading and fine-tuning. During the experiments, TensorBoard was used to visually monitor the training loss, validation accuracy, and disturbance robustness metrics. Matplotlib 3.11 and Seaborn 0.13 were further used for the statistical visualization and analysis of experimental results and ablation studies.

For hyperparameter settings, the dataset was strictly split in chronological order, with the training, validation, and test sets accounting for 70%, 15%, and 15% of the data, respectively, to rigorously prevent future information leakage. Specifically, the training set spans from 1 May 2024 to 15 August 2024, the validation set from 16 August 2024 to 7 September 2024, and the test set from 8 September 2024 to 15 October 2024. To preserve temporal continuity, a time-aware forward-chaining cross-validation strategy was adopted during the training and validation phases, ensuring that only strictly historical data were used to validate future steps. For the final performance evaluation, the model was tested exclusively on the untouched chronological test set. To reliably report statistical variance and enhance the generalization of the experimental results, the training process was repeated 5 times using different random initialization seeds under this exact chronological split, and the final results were reported as the average of these independent runs. During model training, the time-window length was set to T=30, the prediction horizon was set to H=7, and the batch size was set to 64. AdamW was adopted as the optimizer, with an initial learning rate of $\alpha =1\times 10^{-4}$ and a weight decay coefficient of $\lambda =1\times 10^{-5}$. The number of Transformer encoder layers was set to 4, the number of multi-head attention heads was set to 8, the hidden dimension was set to 256, and the dropout ratio was set to 0.2. GELU was adopted as the activation function. The number of training epochs was set to 100, and an early stopping strategy was adopted. Training was terminated early when the validation loss did not decrease for 10 consecutive epochs, thereby preventing overfitting.

#### 4.1.2. Baseline Models and Evaluation Metrics

In the experimental section, ARIMA [52], XGBoost [53], LightGBM [54], LSTM [5], TCN [55], Transformer [6], Attention Fusion [56], Multimodal Transformer [57], Informer [58], TimesNet [59], and Ct-PatchTST [60] were selected as comparison methods. ARIMA (AutoRegressive Integrated Moving Average) is a classical statistical time-series model that establishes a linear relationship between historical sequences and future states through autoregressive, differencing, and moving-average terms. Its advantage lies in its simple model structure and strong interpretability. XGBoost (Extreme Gradient Boosting) is an ensemble learning model based on gradient-boosted decision trees, where residuals are iteratively optimized to improve prediction performance. It can effectively handle nonlinear feature relationships and has strong generalization capability. LightGBM (Light Gradient Boosting Machine) is an efficient tree-based model further optimized within the gradient boosting framework. By using a leaf-wise growth strategy, the training complexity can be reduced, making it suitable for large-scale high-dimensional data scenarios. LSTM (Long Short-Term Memory) is a recurrent neural network based on gating mechanisms, where temporal information flow is controlled through input, forget, and output gates. Its advantage lies in effective modeling of long-term dependencies. TCN (Temporal Convolutional Network) is a temporal modeling network based on causal and dilated convolutions. It captures multiscale temporal features by expanding the receptive field and has advantages in training stability and parallel computation efficiency. Transformer is a sequence modeling architecture based on the self-attention mechanism, which enables dynamic feature learning by computing global dependencies among different temporal positions. Attention Fusion is a multisource feature fusion method based on attention weight allocation, where feature collaborative modeling is achieved by dynamically adjusting the importance of different modalities. Its advantage lies in enhancing the model’s perception of key modalities. Multimodal Transformer further introduces cross-modal attention into the fusion process of multisource heterogeneous signals, enabling deep information interaction among different modalities. As a result, cross-modal associations and global contextual dependencies can be jointly learned, thereby improving fused representation capability in complex scenarios. Informer is a long-sequence time-series forecasting model that reduces the time complexity of self-attention through a ProbSparse mechanism, allowing for efficient processing of extended temporal dependencies. TimesNet transforms one-dimensional time series into two-dimensional tensors to effectively capture multi-periodic temporal variations in complex environments. Ct-PatchTST is an advanced patch-based Transformer architecture tailored for time-series forecasting, utilizing channel-independent patching to preserve local semantic information and improve robustness against noise.

To ensure a strictly fair and rigorous comparison, all baseline models were evaluated under identical experimental conditions. Specifically, every model utilized the exact same chronological data split for training, validation, and testing. They were all configured with the same input window length of 30 and the identical prediction horizon of 7. Furthermore, all models were subjected to the same missing-sensor simulation protocol during the evaluation phase, and their respective hyperparameters were uniformly optimized using grid search on the same validation dataset. The detailed configurations, including input modalities, tuning strategies, evaluation protocols, and the main hyperparameter search spaces for each method, are comprehensively summarized in Table 4.

Based on the precise task definitions established above, different sets of evaluation metrics were employed and mapped to their corresponding objectives. To measure the prediction accuracy of the regression task targeting the future service availability score, MAE (Mean Absolute Error), RMSE (Root Mean Square Error), MAPE (Mean Absolute Percentage Error), and the coefficient of determination $R^{2}$ were utilized. In the disturbance robustness experiment, Disturbance-MAE or Event-RMSE was additionally adopted to measure the regression prediction errors of the service availability score specifically within state transition windows. For sensor-missing scenarios, performance degradation under different missing rates was also analyzed. The mathematical definitions of the evaluation metrics are given as follows:

(32) $$\mathrm{MAE}=\frac{1}{N}\sum_{i=1}^{N}\left|y_{i}-{\hat{y}}_{i}\right|,$$

(33) $$\mathrm{RMSE}=\sqrt{\frac{1}{N}\sum_{i=1}^{N}{\left(y_{i}-{\hat{y}}_{i}\right)}^{2}},$$

(34) $$\mathrm{MAPE}=\frac{100\%}{N}\sum_{i=1}^{N}\left|\frac{y_{i}-{\hat{y}}_{i}}{y_{i}}\right|,$$

(35) $$R^{2}=1-\frac{\sum_{i=1}^{N}{\left(y_{i}-{\hat{y}}_{i}\right)}^{2}}{\sum_{i=1}^{N}{\left(y_{i}-\bar{y}\right)}^{2}},$$

(36) $$\mathrm{AUC}=\int_{0}^{1}\mathrm{TPR}\left(FPR\right)dFPR,$$

where $y_{i}$ denotes the true value, ${\hat{y}}_{i}$ denotes the predicted value, $\bar{y}$ denotes the mean of the true values, and *N* denotes the number of samples.

### 4.2. Overall Performance Comparison

This experiment was designed to verify the overall effectiveness of the proposed method in the state prediction task of complex digital systems. By comparing it with traditional statistical models, machine learning models, deep temporal models, and multisource fusion models, the ability of different modeling paradigms to utilize multisource hardware sensing signals, network communication states, device operational states, and historical output sequences was evaluated.

To rigorously evaluate the statistical significance of the performance improvements, all experiments were repeated five times using different random seeds. The results reported in the tables represent the mean and standard deviation across these independent runs, thereby providing a clear estimation of model variance. Furthermore, to definitively confirm the observed gains, a Wilcoxon signed-rank test was conducted to compare the proposed method against the strongest baseline model, Ct-PatchTST. It is important to clarify that this significance test was not computed on the aggregated macro-metrics of the five runs, but rather on the paired absolute prediction errors of all individual samples across the entire test set. The statistical analysis yields specific *p*-values strictly less than 0.05 across all major evaluation metrics, as detailed in the bottom row of Table 5. This verifies that the performance improvements achieved by our multisource hardware sensing signal fusion network are statistically significant at the sample level and highly robust, rather than a product of random initialization variance over time blocks or disturbance events.

**Figure 4:**
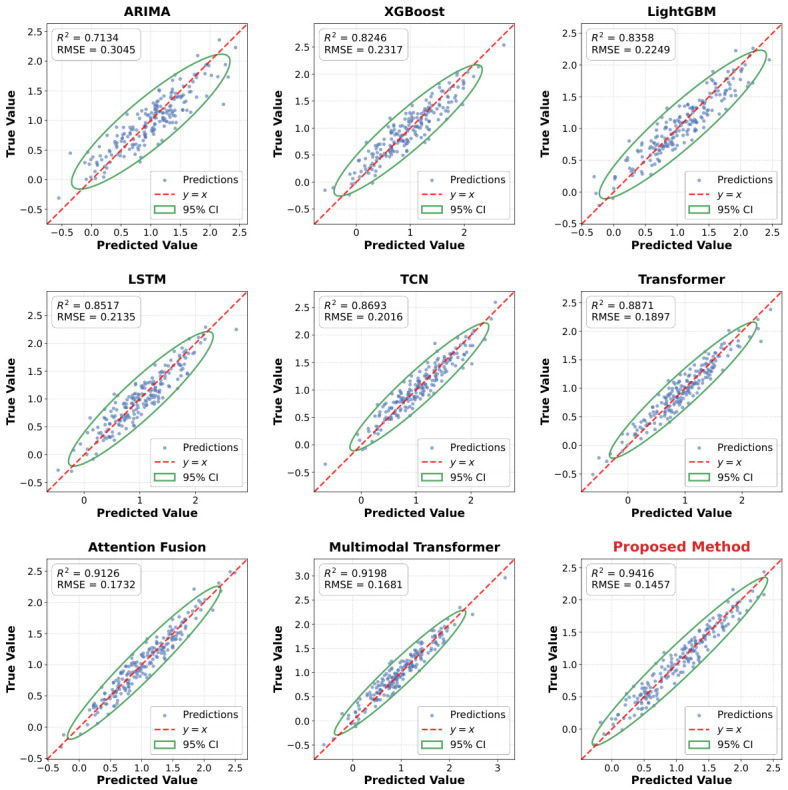
Regression comparison between predicted and true values for different methods, demonstrating that the proposed method produces predictions closest to the ideal y=x line with the highest $R^{2}$ and lowest RMSE.

As shown in Table 5 and Figure 4, the MAE, RMSE, and MAPE of ARIMA are 0.2187, 0.3045, and 18.62%, respectively, while its $R^{2}$ is only 0.7134. This result indicates that the linear autoregressive structure is insufficient for characterizing nonlinear variations and multisource coupling relationships in complex systems. Compared with ARIMA, XGBoost and LightGBM achieve substantial improvements. In particular, the MAE of LightGBM is reduced to 0.1536, and its $R^{2}$ is increased to 0.8358, indicating that tree-based ensemble models can effectively handle nonlinear feature interactions to some extent. However, their capability to model continuous temporal dependencies and cross-source dynamic propagation remains limited. The performance of LSTM and TCN is further improved, suggesting that recurrent memory structures and convolutional temporal structures can capture dynamic variations within historical windows. The RMSE of TCN reaches 0.2016, outperforming LSTM, which reflects that its parallel local convolutional structure provides a more stable representation of multiscale temporal fluctuations.

The results of Transformer, Attention Fusion, and Multimodal Transformer are further improved, indicating that attention mechanisms can establish state dependencies over longer temporal ranges and select more relevant information from multisource signals. The MAE and $R^{2}$ of Multimodal Transformer reach 0.1124 and 0.9198, respectively, which are clearly better than those of the vanilla Transformer. This result demonstrates that multisource representation learning is necessary for state prediction in complex systems. However, these methods still generally assume that different signal sources can be directly aligned within the same time window, and explicit constraints on abrupt disturbances and sensor reliability variations are lacking. The proposed method achieves the best performance, with MAE, RMSE, MAPE, and $R^{2}$ reaching 0.0968, 0.1457, 8.12%, and 0.9416, respectively. This improvement can be attributed to the fact that multisource state prediction is decomposed into three modeling processes: cross-source dynamic alignment, disturbance residual correction, and context-adaptive fusion. The cross-source alignment mechanism can alleviate asynchronous responses among different sensing signals, the disturbance correction structure can separate stable trends from short-term shocks, and the adaptive fusion mechanism can dynamically adjust source contributions according to object states and signal reliability. Therefore, nonlinear temporal dependencies can be learned, while the dynamic propagation and scenario variations of multisource signals in complex digital systems can also be captured, resulting in lower prediction errors and stronger interpretability.

### 4.3. Robustness Comparison

This experiment was designed to verify the prediction robustness of different models in stable operation windows, disturbance occurrence windows, and disturbance recovery windows. To clarify the evaluation protocol, the disturbance window is strictly defined as the start and end time of the controlled interference, denoted as $\left[t_{start},t_{end}\right]$, and the recovery window is defined as $\left[t_{end},t_{end}+\Delta t\right]$. The focus was placed on the error variations of the models under load surges, network congestion, sensor fluctuations, and external event shocks.

**Figure 5:**
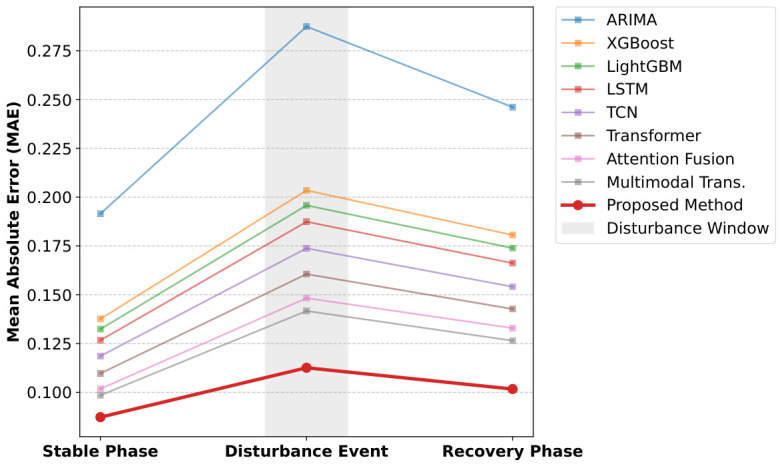
Robustness comparison of different methods across stable, disturbance, and recovery phases, where the proposed method maintains the lowest MAE under non-stationary disturbance conditions.

As shown in Table 6, Table 7 and Figure 5, ARIMA achieves a Stable-MAE of 0.1915 under stable windows, but its Event-MAE increases to 0.2874 under disturbed windows, with an Avg. Drop of 50.08%. This result indicates that its linear autoregressive assumption is difficult to adapt to non-stationary state transitions. XGBoost and LightGBM show clear improvements over ARIMA, with Event-MAE values reduced to 0.2035 and 0.1958, respectively. This demonstrates that tree-based models can fit some nonlinear disturbance features through splitting rules. However, continuous temporal memory mechanisms are still inherently lacking, and relatively large errors remain during the disturbance recovery stage. LSTM and TCN further reduce the errors in both stable and event windows. In particular, the Event-RMSE of TCN is 0.2462, which is lower than that of LSTM, indicating that the convolutional temporal receptive field is more effective in capturing local state transitions.

Transformer, Attention Fusion, and Multimodal Transformer continue to improve the robustness metrics. Among them, the Event-MAE and Recovery-MAE of Multimodal Transformer reach 0.1417 and 0.1265, respectively, indicating that long-range attention and multisource feature interactions can better exploit environmental, device, network, and historical-state information. Nevertheless, disturbance factors are usually implicitly incorporated into a unified representation in these models, which may cause short-term shocks and long-term trends to be mixed during modeling. As a result, the Avg. Drop of Multimodal Transformer remains 43.86%. The proposed method achieves the best results, with Stable-MAE, Event-MAE, and Recovery-MAE reaching 0.0873, 0.1126, and 0.1017, respectively, while the Avg. Drop is reduced to 28.98%. From the perspective of model mechanisms, asynchronous fusion noise among sensing signals can be reduced through cross-source alignment, stable trends and short-term disturbances can be separated through disturbance-aware residual correction, and the influence intensity of the disturbance branch can be controlled through the gating mechanism. Therefore, stable prediction can be maintained under normal conditions, while abrupt deviations can be dynamically compensated under abnormal conditions, significantly improving the robustness of state prediction in complex digital systems.

To systematically verify the generalization capability of the proposed model against unseen disturbances, a leave-one-event-type-out testing protocol was conducted. Under this rigorous evaluation scheme, the model was trained on all disturbance categories except one, and its robustness was subsequently tested exclusively on the withheld event type. The empirical results demonstrate that the model successfully generalizes to unfamiliar disturbance patterns rather than merely memorizing training occurrences. For instance, when heat restriction events were entirely excluded from the training phase, the model achieved an unseen Event-MAE of 0.1195, maintaining a stable performance margin. Similarly, evaluating the model on completely unseen network disconnections yielded an Event-MAE of 0.1234. These findings quantitatively confirm that the disturbance-aware residual correction module effectively learns the fundamental physical representations of state deviations, thereby ensuring high reliability and adaptive robustness in unpredictable industrial digital environments.

### 4.4. Ablation Study

This experiment was designed to verify the actual contribution of each core module in the proposed method to state prediction performance in complex digital systems. The effectiveness of cross-source alignment, time-offset modeling, disturbance correction, adaptive fusion, and reliability constraints was further analyzed.

**Figure 6:**
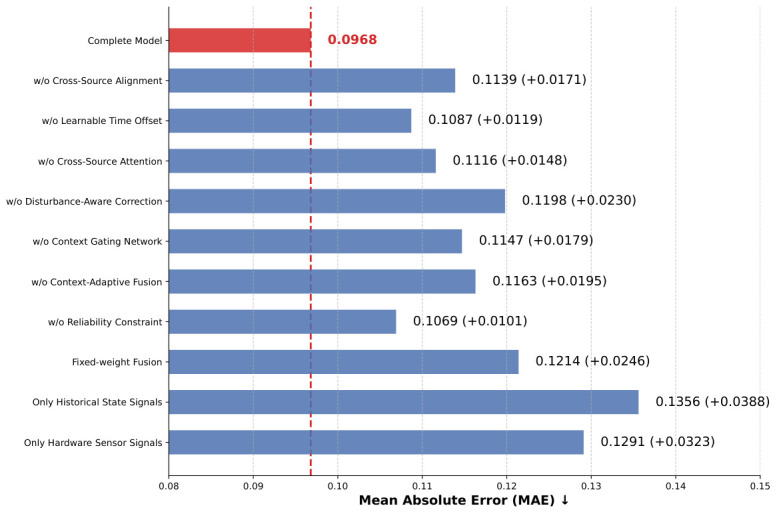
Ablation comparison of MAE values for different model variants, showing that the complete model achieves the lowest prediction error among all variants.

As shown in the Table 8 and Figure 6, the full model achieves the best results, with MAE, RMSE, MAPE, and $R^{2}$ reaching 0.0968, 0.1457, 8.12%, and 0.9416, respectively. This result indicates that the collaborative effect of all modules can form a more stable system-state representation. When the Cross-Source Sensor Signal Alignment Module is removed, the MAE increases to 0.1139 and the $R^{2}$ decreases to 0.9183, indicating that asynchronous responses and dynamic dependencies indeed exist among multisource sensing signals, and simple fusion may introduce alignment errors. After the Learnable Time Offset Mechanism is removed, performance also declines noticeably, suggesting that leading and lagging relationships among different sensing sources are important for state prediction. When Cross-Source Attention is removed, the model becomes less capable of selecting key sensing channels according to the prediction objective, causing the MAE to increase to 0.1116. The most obvious performance degradation occurs when the Disturbance-Aware Residual Correction Module is removed, with the MAE increasing to 0.1198 and the MAPE reaching 10.05%, demonstrating that disturbance modeling is critical for handling abrupt state transitions and non-stationary variations.

Further observations of the fusion-related variants show that the MAE increases to 0.1147 after the Context Gating Network is removed, and to 0.1163 after the Context-Adaptive Multisource Fusion Module is removed. This indicates that fixed or weakly conditioned fusion is difficult to adapt to dynamic signal contributions under different objects, operational stages, and sensor states. When the Reliability Constraint is removed, the model still outperforms most variants, but the MAE increases to 0.1069, suggesting that sensor missingness, noise enhancement, and local anomalies affect the reliability estimation of fusion weights. The performance of Fixed-weight Fusion further decreases, with an $R^{2}$ of only 0.9017, indicating that fixed weights cannot represent the contribution relationships among environmental, device, network, and historical states that vary with context. The results of Only Historical State Signals and Only Hardware Sensor Signals are 0.1356 and 0.1291, respectively, both of which are clearly weaker than the full model. This demonstrates that a single information source cannot fully characterize system-state variations. From the perspective of mathematical modeling characteristics, heterogeneous signal mismatch can be reduced through cross-source dynamic mapping, long-term trends and short-term disturbances can be separated through the residual structure, and the optimal fusion distribution under different contexts can be approximated through the conditional weighting mechanism. Therefore, representation bias caused by static concatenation, single-source modeling, and fixed fusion can be reduced, leading to improved prediction accuracy and generalization capability.

### 4.5. Interpretability and Case Study

To quantitatively validate the interpretability of the context-adaptive multisource fusion module and verify whether the model attends to the physically relevant sources during specific disturbances, a case study tracking the source-weight trajectories was conducted. In this experiment, the dynamic fusion weights assigned to the environmental sensing, device operation, and network communication branches were extracted and analyzed across a chronological window that included two distinct controlled anomaly events, namely a network congestion event and a heat restriction event.

**Figure 7:**
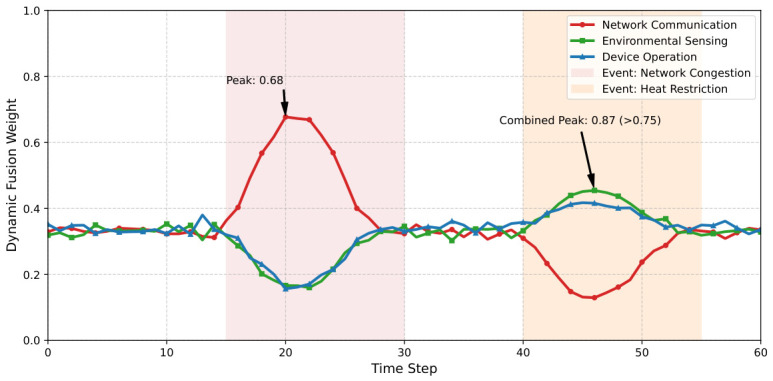
Context-adaptive source-weight trajectories during network congestion and heat restriction events.

As depicted in Figure 7, during the stable operation phase spanning from time step 0 to 14, the fusion weights remain relatively balanced across all sensing modalities, indicating a stable multi-sensor consensus. However, at time step 15, when the controlled network congestion event is triggered, the dynamic weight assigned to the network communication branch experiences a sharp surge, peaking at 0.68, while the weights for environmental and device operation signals correspondingly decline. This confirms that the model correctly identifies the network channels as the primary source of anomaly information. Subsequently, during the heat restriction event introduced at time step 40, a distinctly different attention pattern emerges. The weight for the environmental sensing branch, which captures ambient temperature, alongside the device operation weight tracking internal hardware thermal states, simultaneously rise to dominate the fusion process, reaching combined values exceeding 0.75. These quantitative trajectory shifts provide strong associative interpretability, demonstrating that the proposed adaptive scoring mechanism effectively aligns its mathematical fusion strategy with the underlying physical reality of the industrial edge system.

### 4.6. Discussion

The experimental results demonstrate that the proposed method has strong engineering applicability in the state prediction task of complex industrial and financial–industrial digital systems. Its advantages are reflected not only in the reduction of overall prediction errors but also in its adaptability to non-stationary disturbances in real production and infrastructure scenarios. Taking the edge production test platform of an intelligent equipment manufacturing enterprise in Shijiazhuang, Hebei Province, as an example, edge-computing nodes in the production workshop are required to simultaneously undertake device status monitoring, local data processing, task scheduling, and abnormal alarm functions. During actual operation, the system state is not affected by a single factor, but is jointly determined by cabinet temperature and humidity, power consumption of edge devices, CPU and GPU loads, network response latency, device vibration, and task execution status. Similar infrastructure-level indicators can also be observed in financial–industrial systems, where computing load, communication latency, abnormal connection behaviors, and service availability jointly affect transaction-processing stability. When the workshop temperature rises or cabinet heat dissipation is restricted, device temperature and power consumption may gradually increase. When a certain production task enters a high-load stage, the computational resource utilization and response latency of edge nodes may increase simultaneously. When switch ports suffer congestion or short-term packet loss, task completion rate and service availability may fluctuate significantly. Therefore, state prediction in complex digital systems must jointly consider the dynamic propagation relationships among environmental conditions, device operation, network communication, and system outputs, rather than relying only on historical state sequences for extrapolation.

From the perspective of practical application, the proposed method can provide more reliable state warning and operational decision support for enterprise digital production systems and finance-related digital infrastructure systems. In the stable operation stage, future task completion rate, response time, and service availability can be continuously predicted according to historical states, device loads, and electrical signals, thereby assisting maintenance personnel in determining whether the system remains within a safe operational range. During disturbance occurrence, such as network bandwidth limitation, edge-node load surge, abnormal heat dissipation, or increased device vibration, the disturbance-aware residual correction mechanism enables the model to identify the influence of short-term shocks on system outputs and prevents abrupt anomalies from being misinterpreted as long-term trends. This is of direct value for production sites and financial–industrial service infrastructures, because maintenance personnel need not only to determine whether the current system is abnormal, but also to anticipate whether the anomaly will further affect task execution efficiency, transaction-service stability, or service quality. Meanwhile, the signal contribution weights output by the context-adaptive fusion module can help analyze key influencing factors under different scenarios. For example, increased weights of environmental sensing and device temperature signals during high-temperature periods indicate that heat dissipation conditions are the primary source of risk. Increased weights of network communication states during congestion windows suggest that switches, link bandwidth, or edge-node communication interfaces should be prioritized for inspection. Therefore, the proposed method not only improves prediction accuracy but also strengthens the interpretable connection between model outputs and engineering operations, enabling multisource hardware sensing data to effectively support state monitoring, risk warning, and on-site decision-making in complex industrial and financial–industrial digital systems.

### 4.7. Limitation and Future Work

Several limitations remain in this study that must be explicitly acknowledged. First, the dataset was exclusively collected from a specific edge-computing and digital production test platform of a single intelligent equipment manufacturing enterprise in Hebei Province. Although multisource hardware sensing signals, including environmental, electrical, vibration, network communication, and system-output signals, were covered, the industrial scenario remains highly concentrated. The generalization capability of the model in broader and more diverse types of complex digital systems remains limited and requires further verification. Second, the current empirical validation relies heavily on controllable experiments used to construct disturbance events, such as triggered load surges, network congestion, restricted heat dissipation, and short-term disconnections. In contrast, anomaly types in independent, real-world industrial sites are vastly more complex and unpredictable. These include random failure modes such as long-term equipment aging, sensor measurement drift, cross-system cascading failures, and unrecorded human operation errors, which have not yet been fully captured in the current controlled experimental setup. In addition, the proposed method contains multiple modules, including cross-source alignment, disturbance residual correction, and context-adaptive fusion. Although prediction accuracy and robustness are improved, computational overhead and deployment complexity are correspondingly increased.

Future work will be rigorously carried out from the perspectives of large-scale deployment validation, data expansion, and model lightweighting. Crucially, future validation must be conducted on multiple independent, real-world industrial deployments to test the framework against highly random and compound failure modes. Multisource sensing data will be continuously collected from broader enterprise domains and entirely different types of edge systems to critically evaluate the adaptability of the model across industries, devices, and highly non-stationary operating environments. On the other hand, the number of model parameters will be further reduced by combining pruning, distillation, and low-rank decomposition, so that the model can be more suitable for deployment on resource-constrained edge terminals. Meanwhile, online learning mechanisms will also be incorporated in future work, allowing the model to continuously update according to newly emerging device states and unforeseen disturbance patterns, thereby improving its stability and practical value in long-term, unconstrained operation scenarios.

## 5. Conclusions

With the rapid development of intelligent manufacturing, edge computing, and industrial and financial–industrial digital systems, large volumes of multisource hardware sensing signals are continuously generated in complex production environments from environmental conditions, electrical states, vibration responses, network communication, and device operational status. How to extract stable state representations from such heterogeneous, asynchronous, and disturbance-sensitive sensing data using artificial intelligence methods and further achieve accurate, robust, and interpretable system state prediction has become an important issue in the field of AI-driven sensing. For the specific edge-computing and digital production test scenario of an intelligent equipment manufacturing enterprise in Hebei Province, China, a multisource hardware sensing signal fusion network is proposed in this study. Environmental sensing, device power, edge-node operation, vibration monitoring, network communication, and system output states are uniformly modeled as multisource engineering sensing signals, thereby providing effective data-driven support for operation monitoring and intelligent decision-making within the investigated types of industrial digital systems and related financial–industrial infrastructure scenarios.

The main innovation of this study lies in the construction of an end-to-end prediction framework composed of cross-source sensing signal alignment, disturbance-aware residual correction, and context-adaptive fusion. The cross-source sensing signal alignment module is used to characterize leading, lagging, and collaborative response relationships among different sensing sources, which strongly facilitates temporal coherence. The disturbance-aware residual correction module is designed to separate stable operational trends from abrupt disturbance effects, thereby substantially mitigating disturbance contamination. The context-adaptive fusion module dynamically adjusts the contribution of different signal sources according to object states, sensor reliability, and disturbance intensity. Experimental results validated under the considered industrial test platform show that the proposed method achieves highly competitive performance in the overall state prediction task, with MAE, RMSE, MAPE, and $R^{2}$ reaching 0.0968, 0.1457, 8.12%, and 0.9416, respectively, outperforming baseline methods including ARIMA, XGBoost, LightGBM, LSTM, TCN, Transformer, Attention Fusion, and Multimodal Transformer. In the disturbance robustness experiment, the Event-MAE and Event-RMSE of the proposed method are reduced to 0.1126 and 0.1694, respectively, with an Avg. Drop of only 28.98%, indicating that the method can respond more stably to non-stationary scenarios such as load surges, network congestion, and device anomalies. Overall, an effective engineering-oriented modeling scheme is provided for AI-driven multisource sensing and robust state prediction under the specific constraints and configurations of the tested industrial edge platform, with potential applicability to finance-related digital infrastructure monitoring.

## Figures and Tables

**Table 1:** Collection summary of multisource hardware sensing data used in this study.

Data Type	Sensor/Device Model	Data Volume
Environmental sensing data	Sensirion SHT31, BH1750, Bosch BMP280	263,520 records
Equipment power data	INA219 voltage–current sensor	3,162,240 records
Edge-device operational status data	NVIDIA Jetson Xavier NX, Raspberry Pi 4B	3,162,240 records
Equipment vibration data	ADXL345 three-axis accelerometer	31,622,400 segments
Network communication status data	TP-Link TL-SG108E, Raspberry Pi network probe	1,581,120 records

**Table 2:** Detailed list of multisource hardware sensing variables, physical units, and original sampling rates.

Category	Variable Name	Physical Unit	Original Sampling Rate
Environmental	Temperature	Celsius	1 min
Environmental	Relative Humidity	Percentage	1 min
Environmental	Illumination	Lux	1 min
Environmental	Barometric Pressure	hPa	1 min
Electrical	Voltage	V	5 s
Electrical	Current	A	5 s
Electrical	Power Consumption	W	5 s
Operational	CPU Load	Percentage	5 s
Operational	GPU Load	Percentage	5 s
Operational	Memory Usage	Percentage	5 s
Operational	Device Temperature	Celsius	5 s
Vibration	X/Y/Z Acceleration	g	50 Hz
Vibration	Vibration Amplitude	mm/s	50 Hz
Network	Uplink/Downlink Traffic	Mbps	10 s
Network	Request Frequency	Hz	10 s
Network	Average Response Latency	ms	10 s
Network	Packet Loss Rate	Percentage	10 s

**Table 3:** Distribution of normal and abnormal samples, categories, and corresponding disturbance types.

State	Category	Disturbance Type	Number of Samples
Normal	Stable Operation	None	233,215
Abnormal	High Equipment Load	Edge-computing load surges	8500
Abnormal	Network Congestion	Bandwidth limitations	8000
Abnormal	Heat Dissipation Restriction	Restricted heat dissipation events	5500
Abnormal	Communication Failure	Short-term network disconnections	4500
Abnormal	Environmental Anomaly	Environmental temperature–humidity fluctuations	3805

**Table 4:** Baseline configuration specifying input modalities, tuning strategy, evaluation protocol, and main hyperparameters.

Method	Input Modalities	Tuning Strategy	Evaluation Protocol	Main Hyperparameters
ARIMA	All fused sensors	Grid Search	T=30, H=7	p∈{1, 2, 3}, d∈{0, 1}, q∈{1, 2, 3}
XGBoost	All fused sensors	Grid Search	T=30, H=7	max_depth ∈{3, 5, 7}, n_estimators ∈{50,100,200}
LightGBM	All fused sensors	Grid Search	T=30, H=7	num_leaves ∈{31,63}, learning_rate ∈{0.01,0.05,0.1}
LSTM	All fused sensors	Grid Search	T=30, H=7	hidden_size ∈{64,128}, num_layers ∈{1,2,3}
TCN	All fused sensors	Grid Search	T=30, H=7	kernel_size ∈{3,5}, dropout ∈{0.1,0.2}
Transformer	All fused sensors	Grid Search	T=30, H=7	d_model ∈{128,256}, n_heads ∈{4,8}
Attention Fusion	All fused sensors	Grid Search	T=30, H=7	attention_dim ∈{64,128}, learning_rate ∈{1e−4,1e−3}
Multimodal Transformer	All fused sensors	Grid Search	T=30, H=7	cross_heads ∈{4,8}, e_layers ∈{2,3}
Informer	All fused sensors	Grid Search	T=30, H=7	factor ∈{3,5}, d_model ∈{128,256}
TimesNet	All fused sensors	Grid Search	T=30, H=7	top_k ∈{3,5}, d_ff ∈{256,512}
Ct-PatchTST	All fused sensors	Grid Search	T=30, H=7	patch_len ∈{8,16}, stride ∈{8,16}
Proposed method	All fused sensors	Grid Search	T=30, H=7	d_model ∈{128,256}, λ∈{0.1,0.5,1.0}

**Table 5:** Overall performance comparison of different methods on the state prediction task of complex digital systems.

Method	MAE ↓	RMSE ↓	MAPE (%) ↓	$R^{2}↑$
ARIMA	0.2187±0.0052	0.3045±0.0061	18.62±0.35	0.7134±0.0085
XGBoost	0.1598±0.0038	0.2317±0.0042	13.84±0.28	0.8246±0.0062
LightGBM	0.1536±0.0035	0.2249±0.0039	13.27±0.25	0.8358±0.0058
LSTM	0.1462±0.0029	0.2135±0.0031	12.64±0.22	0.8517±0.0045
TCN	0.1378±0.0025	0.2016±0.0028	11.86±0.19	0.8693±0.0041
Transformer	0.1286±0.0021	0.1897±0.0024	10.96±0.16	0.8871±0.0036
Attention Fusion	0.1169±0.0019	0.1732±0.0022	9.87±0.14	0.9126±0.0031
Multimodal Transformer	0.1124±0.0017	0.1681±0.0019	9.43±0.13	0.9198±0.0028
Informer	0.1085±0.0015	0.1623±0.0017	9.15±0.12	0.9234±0.0025
TimesNet	0.1042±0.0014	0.1558±0.0016	8.86±0.11	0.9305±0.0021
Ct-PatchTST	0.0995±0.0013	0.1492±0.0015	8.45±0.10	0.9372±0.0019
Proposed method	0.0968±0.0011	0.1457±0.0012	8.12±0.08	0.9416±0.0014
*p*-value (vs. Ct-PatchTST)	<0.001	<0.001	<0.001	<0.001

**Table 6:** Robustness comparison of different methods under stable and disturbed windows.

Method	Stable-MAE ↓	Stable-RMSE ↓	Event-MAE ↓	Event-RMSE ↓	Recovery-MAE ↓	Avg. Drop (%) ↓
ARIMA	0.1915±0.0042	0.2668±0.0051	0.2874±0.0068	0.3916±0.0075	0.2461±0.0055	50.08±1.25
XGBoost	0.1376±0.0031	0.1989±0.0038	0.2035±0.0052	0.2894±0.0061	0.1806±0.0042	47.89±1.12
LightGBM	0.1324±0.0028	0.1921±0.0035	0.1958±0.0048	0.2763±0.0056	0.1739±0.0039	47.88±1.08
LSTM	0.1267±0.0025	0.1845±0.0029	0.1874±0.0041	0.2648±0.0049	0.1662±0.0034	47.91±1.02
TCN	0.1186±0.0022	0.1728±0.0026	0.1738±0.0037	0.2462±0.0043	0.1541±0.0029	46.54±0.95
Transformer	0.1097±0.0019	0.1615±0.0022	0.1606±0.0032	0.2293±0.0038	0.1427±0.0025	46.40±0.88
Attention Fusion	0.1018±0.0016	0.1512±0.0019	0.1483±0.0028	0.2136±0.0031	0.1329±0.0021	45.68±0.82
Multimodal Transformer	0.0985±0.0015	0.1464±0.0017	0.1417±0.0025	0.2058±0.0028	0.1265±0.0019	43.86±0.75
Informer	0.0962±0.0014	0.1435±0.0016	0.1354±0.0022	0.1982±0.0025	0.1213±0.0017	40.74±0.68
TimesNet	0.0931±0.0013	0.1392±0.0015	0.1287±0.0019	0.1885±0.0022	0.1146±0.0015	38.23±0.61
Ct-PatchTST	0.0905±0.0012	0.1354±0.0014	0.1215±0.0016	0.1796±0.0019	0.1082±0.0013	34.25±0.54
Proposed method	0.0873±0.0010	0.1318±0.0012	0.1126±0.0014	0.1694±0.0016	0.1017±0.0011	28.98±0.45

**Table 7:** Detailed breakdown of Event-MAE across different disturbance types.

Method	Load Surge MAE ↓	Network Congestion MAE ↓	Heat Restriction MAE ↓	Disconnection MAE ↓
ARIMA	0.2825±0.0071	0.2941±0.0078	0.2798±0.0069	0.2932±0.0076
XGBoost	0.1984±0.0054	0.2112±0.0062	0.1965±0.0051	0.2079±0.0059
LightGBM	0.1912±0.0049	0.2045±0.0055	0.1887±0.0046	0.1988±0.0051
LSTM	0.1834±0.0042	0.1956±0.0048	0.1792±0.0040	0.1914±0.0045
TCN	0.1702±0.0038	0.1815±0.0044	0.1668±0.0036	0.1767±0.0041
Transformer	0.1581±0.0033	0.1664±0.0039	0.1542±0.0031	0.1637±0.0036
Attention Fusion	0.1455±0.0029	0.1538±0.0034	0.1412±0.0027	0.1527±0.0032
Multimodal Transformer	0.1386±0.0026	0.1472±0.0031	0.1351±0.0024	0.1459±0.0029
Informer	0.1325±0.0024	0.1408±0.0027	0.1296±0.0022	0.1387±0.0025
TimesNet	0.1261±0.0021	0.1335±0.0024	0.1234±0.0019	0.1318±0.0022
Ct-PatchTST	0.1192±0.0018	0.1264±0.0021	0.1165±0.0017	0.1239±0.0019
Proposed method	0.1105±0.0015	0.1168±0.0018	0.1084±0.0014	0.1147±0.0016

**Table 8:** Ablation study results of the proposed method.

Model Variant	MAE ↓	RMSE ↓	MAPE (%) ↓	$R^{2}↑$
Full model	0.0968±0.0011	0.1457±0.0012	8.12±0.08	0.9416±0.0014
w/o Cross-Source Sensor Signal Alignment Module	0.1139±0.0018	0.1686±0.0021	9.58±0.14	0.9183±0.0022
w/o Learnable Time Offset Mechanism	0.1087±0.0015	0.1609±0.0018	9.17±0.12	0.9271±0.0019
w/o Cross-Source Attention	0.1116±0.0016	0.1648±0.0019	9.36±0.13	0.9225±0.0021
w/o Disturbance-Aware Residual Correction Module	0.1198±0.0021	0.1764±0.0024	10.05±0.17	0.9079±0.0026
w/o Context Gating Network	0.1147±0.0019	0.1702±0.0022	9.72±0.15	0.9156±0.0023
w/o Context-Adaptive Multisource Fusion Module	0.1163±0.0020	0.1728±0.0023	9.84±0.16	0.9124±0.0024
w/o Reliability Constraint	0.1069±0.0014	0.1587±0.0016	9.01±0.11	0.9295±0.0017
Fixed-weight Fusion	0.1214±0.0022	0.1812±0.0026	10.31±0.18	0.9017±0.0028
Only Historical State Signals	0.1356±0.0028	0.1983±0.0033	11.49±0.23	0.8738±0.0036
Only Hardware Sensor Signals	0.1291±0.0025	0.1907±0.0029	10.92±0.20	0.8864±0.0032

## Data Availability

The data presented in this study are available upon request from the corresponding author.

## References

[1] Shi L., Tian M., Yi Y., Hu X., Wang X., Yang Y., Li M. (2026). Artificial Intelligence-Driven Multimodal Sensor Fusion for Complex Market Systems via Federated Transformer-Based Learning. Sensors.

[2] Zhang Y., Fu C., Wang X., Zhang Y., Xiong Z., Pan J., Yin J. (2026). AI-Driven Sensing for Cross-Lingual Risk Prediction via Semantic Alignment and Multimodal Temporal Fusion. Appl. Sci..

[3] Liu F.T., Ting K.M., Zhou Z.H. (2008). Isolation forest. Proceedings of the 2008 Eighth IEEE International Conference on Data Mining.

[4] Schölkopf B., Williamson R.C., Smola A., Shawe-Taylor J., Platt J. (1999). Support vector method for novelty detection. Adv. Neural Inf. Process. Syst..

[5] Hochreiter S., Schmidhuber J. (1997). Long short-term memory. Neural Comput..

[6] Vaswani A., Shazeer N., Parmar N., Uszkoreit J., Jones L., Gomez A.N., Kaiser Ł., Polosukhin I. (2017). Attention is all you need. Adv. Neural Inf. Process. Syst..

[7] Ahmad M., Rehman A. (2025). Multi-Source Information Fusion for Anomaly Detection in Smart Grids Using Federated Learning. Chin. J. Inf. Fusion.

[8] Wang C., Wang H., Guo J., Li W., Jiang Q. (2025). Multimodal Sentiment-Aware Volatility Forecasting: A Deep Fusion Approach with Social Media Data. Proceedings of the 2025 International Conference on Generative Artificial Intelligence for Business.

[9] Araci D. (2019). Finbert: Financial sentiment analysis with pre-trained language models. arXiv.

[10] Asadi N., Fatemeh Ghoreishi S. (2024). Dynamic sensor selection for efficient monitoring of coupled multidisciplinary systems. J. Comput. Inf. Sci. Eng..

[11] Tabatabaie M., He S. (2024). Driver maneuver interaction identification with anomaly-aware federated learning on heterogeneous feature representations. Proc. ACM Interact. Mob. Wearable Ubiquitous Technol..

[12] Sun R., Ren Y. (2024). A multi-source heterogeneous data fusion method for intelligent systems in the Internet of Things. Intell. Syst. Appl..

[13] Zigui L., Caluyo F., Hernandez R., Sarmiento J., Rosales C.A. (2024). Improving communication networks to transfer data in real time for environmental monitoring and data collection. Nat. Eng. Sci..

[14] Sun L., Huang X., Wang X., Zhao C., Zhang Y., Wang Y. (2026). Deep learning-based mechanism-and signal-driven framework for early prediction of milling surface roughness. Mech. Syst. Signal Process..

[15] Raja L., Ramalingam S., Sowbarnika S., Vishnupriya V., Saranyanandhini D. (2023). Fault Aware Data Prediction in Wireless Sensor Networks Using Machine Learning. Proceedings of the 2023 International Conference on Sustainable Communication Networks and Application (ICSCNA).

[16] Vakitbilir N., Islam A., Gomez A., Stein K.Y., Froese L., Bergmann T., Sainbhi A.S., McClarty D., Raj R., Zeiler F.A. (2024). Multivariate modelling and prediction of High-Frequency Sensor-Based cerebral physiologic signals: Narrative review of machine learning methodologies. Sensors.

[17] Cao L., Wang J., Su J., Luo Y., Cao Y., Braatz R.D., Gopaluni B. (2025). Comprehensive analysis on machine learning approaches for interpretable and stable soft sensors. IEEE Trans. Instrum. Meas..

[18] Fang W., Qin H., Liu G., Yang X., Xu Z., Jia B., Zhang Q. (2023). A method for spatiotemporally merging multi-source precipitation based on deep learning. Remote Sens..

[19] Mahmoud A., Mohammed A. (2026). Enhancing time series forecasting: A hybrid TCN-transformer approach. Neural Comput. Appl..

[20] Liu H., Wan W., Chen J., Feng Y. (2026). Topology-aware graph neural network for fusing sparse sensor data with finite element predictions in structural random vibration assessment. Expert Syst. Appl..

[21] Song F., Zeng Q., Zhang R., Zhu X., Ye X., Zhang Z. (2025). Multi-UAV Cooperative Navigation Based on Multi-Source Information Fusion: A Review. IEEE Sens. J..

[22] Kannan V., Dao D.V., Li H. (2023). An information fusion approach for increased reliability of condition monitoring with homogeneous and heterogeneous sensor systems. Struct. Health Monit..

[23] Tong X., Lv C., Si J., Wang C., Bao J. (2026). Building resilient digital twin with digital cousins: A method for accurate prediction from multi-source fusion in manufacturing. Int. J. Comput. Integr. Manuf..

[24] Ma L., Yang Q., Peng K. (2025). A unified representation and fusion framework of multi-source heterogeneous data for fault diagnosis in industrial processes. Adv. Eng. Inform..

[25] Xiang X., Li K., Huang B., Cao Y. (2022). A multi-sensor data-fusion method based on cloud model and improved evidence theory. Sensors.

[26] Zhang Y., Zhang B., Shen C., Liu H., Huang J., Tian K., Tang Z. (2024). Review of the field environmental sensing methods based on multi-sensor information fusion technology. Int. J. Agric. Biol. Eng..

[27] Suslu B., Ali F., Jennions I.K. (2023). Understanding the role of sensor optimisation in complex systems. Sensors.

[28] Jianxi Y. (2023). Mathematical modeling method based on heterogeneous cellular network algorithm and computer multi-dimensional space. Meas. Sens..

[29] Lee W.Y., Jovanov L., Philips W. (2022). Cross-modality attention and multimodal fusion transformer for pedestrian detection. Proceedings of the European Conference on Computer Vision.

[30] Agostinho L., Pereira D., Hiolle A., Pinto A. (2024). TEFu-Net: A time-aware late fusion architecture for robust multi-modal ego-motion estimation. Robot. Auton. Syst..

[31] Zhang Y., Zang Z., Zhang X., Song L., Yu Z., Wang Y., Gao Y., Wang L. (2024). Fault diagnosis of industrial robot based on multi-source data fusion and channel attention convolutional neural networks. IEEE Access.

[32] Li R., Sun Z., Liu Y., Liu X. (2025). Multi-Source Data Fusion-Based Synthesis and Correlation Analysis of Power Grid Equipment Events. Proceedings of the 2025 5th International Conference on Intelligent Power and Systems (ICIPS).

[33] Liu X., Liu Y., Wang T. (2025). Performance Improvement for Joint Source and Sensor Localization by Integrating Sensors as Transceivers in Asynchronous Sensor Networks. IEEE Trans. Aerosp. Electron. Syst..

[34] Sun Y., Zheng Y. (2023). A method of gas sensor drift compensation based on intrinsic characteristics of response curve. Sci. Rep..

[35] Liu Q., Wang Y., Zhao F., Zheng C., Xie J. (2025). A Review of the Research Progress of Sensor Monitoring Technology in Harsh Engineering Environments. Sensors.

[36] Feng Z., Hu H., Yang S. (2023). Cross-sensor correlative feature learning and fusion for intelligent fault diagnosis. IEEE Trans. Ind. Inform..

[37] Lin S., Huang F., Lai T., Lai J., Wang H., Weng J. (2024). Robust heterogeneous model fitting for multi-source image correspondences. Int. J. Comput. Vis..

[38] Yu B., Huang X., Xiang H., Yang J., Tu L., Zhang Y., Jiao Y. (2026). A Multi-Source Time-Series Remote Sensing Data Fusion Network for High-Spatial-Resolution Crop Classification. IEEE J. Sel. Top. Appl. Earth Obs. Remote Sens..

[39] Wang Y., Xu Y., Yang J., Chen Z., Wu M., Li X., Xie L. (2023). Sensor alignment for multivariate time-series unsupervised domain adaptation. Proceedings of the AAAI Conference on Artificial Intelligence.

[40] Kuhse D., Holscher N., Gunzel M., Teper H., Von Der Bruggen G., Chen J.J., Lin C.C. (2024). Sync or sink? the robustness of sensor fusion against temporal misalignment. Proceedings of the 2024 IEEE 30th Real-Time and Embedded Technology and Applications Symposium (RTAS).

[41] Werner C., Wallrabe U., Christen A., Comella L.M., Dormann C.F., Göritz A., Grote R., Haberstroh S., Jouda M., Kiese R. (2024). ECOSENSE-Multi-scale quantification and modelling of spatio-temporal dynamics of ecosystem processes by smart autonomous sensor networks. Res. Ideas Outcomes.

[42] Geng X., Jiao L., Li L., Liu F., Liu X., Yang S., Zhang X. (2023). Multisource joint representation learning fusion classification for remote sensing images. IEEE Trans. Geosci. Remote Sens..

[43] Liang J., Chen Y., Wu Y., Miao Z., Zhang H., Wang Y. (2022). Adaptive prescribed performance control of unmanned aerial manipulator with disturbances. IEEE Trans. Autom. Sci. Eng..

[44] Kim J., Kim M.J. (2024). Disturbance-aware model predictive control of underactuated robotics systems. Proceedings of the 2024 IEEE/RSJ International Conference on Intelligent Robots and Systems (IROS).

[45] García-Pérez A. (2022). On robustness for spatio-temporal data. Mathematics.

[46] Cai T., Namkoong H., Yadlowsky S. (2026). Diagnosing model performance under distribution shift. Oper. Res..

[47] Shen H., Zhan J., He T. (2025). RL-enhanced disturbance-aware MPC for fast and robust UAV trajectory tracking. Proceedings of the 2025 IEEE International Conference on Systems, Man, and Cybernetics (SMC).

[48] Phuong N.D., Nguyen T.Q. (2026). High-resolution day-ahead load forecasting under lunar holiday drift using causal wavelet decomposition and event-aware masked learning. Res. Sq..

[49] Dev P.P., Das P., Hazari R. (2026). Cross-modal uncertainty modeling framework for unseen video anomaly detection. Neurocomputing.

[50] Guo L., Li W., Zhu Y., Yu X., Wang Z. (2023). Composite disturbance filtering: A novel state estimation scheme for systems with multisource, heterogeneous, and isomeric disturbances. IEEE Open J. Ind. Electron. Soc..

[51] Turner M.G., Seidl R. (2023). Novel disturbance regimes and ecological responses. Annu. Rev. Ecol. Evol. Syst..

[52] Nerlove M. (1971). Time Series Analysis, Forecasting, and Control.

[53] Chen T., Guestrin C. (2016). Xgboost: A scalable tree boosting system. Proceedings of the 22nd ACM Sigkdd International Conference on Knowledge Discovery and Data Mining.

[54] Ke G., Meng Q., Finley T., Wang T., Chen W., Ma W., Ye Q., Liu T.Y. (2017). Lightgbm: A highly efficient gradient boosting decision tree. Adv. Neural Inf. Process. Syst..

[55] Bai S., Kolter J.Z., Koltun V. (2018). An empirical evaluation of generic convolutional and recurrent networks for sequence modeling. arXiv.

[56] Bahdanau D., Cho K., Bengio Y. (2014). Neural machine translation by jointly learning to align and translate. arXiv.

[57] Lu J., Batra D., Parikh D., Lee S. (2019). Vilbert: Pretraining task-agnostic visiolinguistic representations for vision-and-language tasks. Adv. Neural Inf. Process. Syst..

[58] Cui Y., Li Z., Wang Y., Dong D., Gu C., Lou X., Zhang P. (2024). Informer model with season-aware block for efficient long-term power time series forecasting. Comput. Electr. Eng..

[59] Cai X. (2025). An attention-enhanced TimesNet time series model for predicting the commodity price. Alex. Eng. J..

[60] Lu K., Huo M., Li Y., Zhu Q., Chen Z. (2025). Ct-patchtst: Channel-time patch time-series transformer for long-term renewable energy forecasting. Proceedings of the 2025 10th International Conference on Computer and Information Processing Technology (ISCIPT).

